# Ion Mobility–Based Enrichment-Free N-Terminomics Analysis Reveals Novel Legumain Substrates in Murine Spleen

**DOI:** 10.1016/j.mcpro.2024.100714

**Published:** 2024-01-08

**Authors:** Alexander R. Ziegler, Antoine Dufour, Nichollas E. Scott, Laura E. Edgington-Mitchell

**Affiliations:** 1 Department of Biochemistry and Pharmacology, Bio21 Molecular Science and Biotechnology Institute, The University of Melbourne, Parkville, Victoria, Australia; 2 Department of Physiology and Pharmacology, University of Calgary, Calgary, Alberta, Canada; 3 McCaig Institute for Bone and Joint Health, University of Calgary, Calgary, Alberta, Canada; 4 Department of Microbiology and Immunology, Peter Doherty Institute, The University of Melbourne, Parkville, Victoria, Australia

**Keywords:** legumain, asparaginyl endopeptidase, N-terminomics, degradomics, protease substrates, FAIMS

## Abstract

Aberrant levels of the asparaginyl endopeptidase legumain have been linked to inflammation, neurodegeneration, and cancer, yet our understanding of this protease is incomplete. Systematic attempts to identify legumain substrates have been previously confined to *in vitro* studies, which fail to mirror physiological conditions and obscure biologically relevant cleavage events. Using high-field asymmetric waveform ion mobility spectrometry (FAIMS), we developed a streamlined approach for proteome and N-terminome analyses without the need for N-termini enrichment. Compared to unfractionated proteomic analysis, we demonstrate FAIMS fractionation improves N-termini identification by >2.5 fold, resulting in the identification of >2882 unique N-termini from limited sample amounts. In murine spleens, this approach identifies 6366 proteins and 2528 unique N-termini, with 235 cleavage events enriched in WT compared to legumain-deficient spleens. Among these, 119 neo-N-termini arose from asparaginyl endopeptidase activities, representing novel putative physiological legumain substrates. The direct cleavage of selected substrates by legumain was confirmed using *in vitro* assays, providing support for the existence of physiologically relevant extra-lysosomal legumain activity. Combined, these data shed critical light on the functions of legumain and demonstrate the utility of FAIMS as an accessible method to improve depth and quality of N-terminomics studies.

## Highlights

• FAIMS improves protein and N-termini coverage using microgram amounts of sample.
• This method allows for enrichment-free identification of N-terminal cleavage events.
• In murine spleen, legumain KO led to altered proteolysis and neutrophil proteins.
• 119 asparagine-specific cuts were lost in KO mice, revealing legumain substrates.
• Substrate profile suggests broad extra-lysosomal functions of legumain.

## Graphical Abstract

**Figure:**
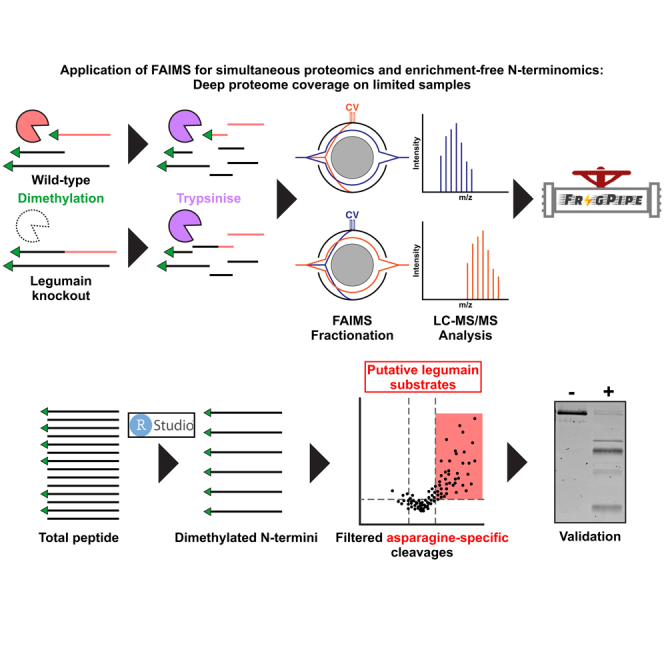

## In Brief

N-terminomics studies traditionally require large amounts of starting material and the use of enrichment methods to isolate N-terminal peptides. Herein we show that by combining protein level labeling and FAIMS fractionation, microgram amounts of protein can yield deep coverage of the neo-N-terminome and protein abundance changes in a single experiment without the need for N-termini enrichment. We applied this method to identify 119 physiologically relevant substrates for the cysteine protease legumain, shedding light on its potential extra-lysosomal functions.

Proteases comprise approximately 3% of the human genome and catalyze the cleavage of peptide bonds (1). Proteolysis is essential for maintaining protein homeostasis, altering substrate structure, function, and localization (2). Proteases contribute to vital cellular functions such as cell growth and repair (3), immune signaling (4), and wound healing (5, 6), and dysregulated protease activities underpin numerous pathological conditions including cancer, inflammation, neurodegeneration, and gastrointestinal diseases (7). Knowledge of specific cleavage events is crucial in understanding the mechanistic contributions of proteases to normal physiology and disease. The need for sensitive approaches to catalog these events has led to the development of peptide-centric N-terminomics methods, which have rapidly developed over the last decade (8, 9, 10). Leveraging advancements in liquid chromatography mass spectrometry (LC-MS), peptide-based N-terminomics methods have become the gold standard for identification of protease substrates at scale (11). These techniques provide site-specific resolution of cleavage events and have led to substrate discovery for numerous proteases including matrix metalloproteases (MMP) MMP-2 and MMP-9 (12), caspases (13, 14), ADAMTS7 (15), HTRA1 (16), and cathepsins (17, 18). While N-terminomics techniques have improved the ability to identify biologically important cleavage events, these approaches are not without their limitations.

Current generation N-terminomics methods typically involve the enrichment of N-terminal peptides (N-termini) using either positive or negative enrichment methods (19). These methods uniformly involve tagging the N-terminal α-amines of proteins prior to *in vitro* proteolytic digestion, therefore allowing native and protease-generated (neo) N-termini, which contain a defined chemical tag or native acetylation event, to be differentiated from the unmodified, internal peptides (9). The tagging of α-amines with enrichable chemical handles permits effective positive selection of N-termini, including chemical labeling with biotin (20, 21, 22, 23) or phospho-tags (24) or enzyme-mediated conjugates (*e.g.*, subtiligase) (25, 26). In contrast, negative selection approaches leverage the exposed α-amines of N-termini to allow the depletion of internal peptides using either N-hydroxysuccinimide polymers or resins, such as those used in terminal amine isotopic labeling of substrates (TAILS) (27, 28) or Nrich (29, 30). Alternatively, internal peptides may be hydrophobically tagged as in HYTANE (31) and HUNTER (32) or chromatographically separated as in the COFRADIC method (33, 34), to allow peptide depletion. While both positive and negative selection have proven effective to identify new protease substrates, these enrichment-based approaches have typically required large sample inputs, expensive and specialized materials, and user expertise, all while sacrificing the acquisition of total proteomic information. While multiple N-terminomics studies have sought to address this issue by examining nonenriched samples in parallel (18, 35, 36, 37), the separation of the proteome and the N-terminome can make it difficult to ascertain whether N-termini differences are due to increased cleavage or protein abundance changes. Thus, while powerful, current N-terminomics approaches still provide limited proteomic depth and utilize technologies not accessible to the broader protease and proteomics community.

A widely utilized approach to improve proteome depth is the use of orthogonal chromatographic fractionation prior to LC-MS (38, 39). While a range of chromatographic approaches exist to fractionate proteomics samples, an alternative and increasingly accessible technology is high-field asymmetric waveform ion mobility spectrometry (FAIMS) (40, 41). This technology allows gas phase–based fractionation of peptides following chromatographic separation by filtering ion populations prior to their introduction into the mass spectrometer (42). FAIMS allows for the fractionation of samples without the need for off-line sample handling, which is cumbersome and leads to significant sample loss (43). Moreover, FAIMS is uniquely suited for limited sample amounts, allowing enhanced detection sensitivity (44) and dramatically improved proteomic depth (45). While widely used to improve proteome coverage, FAIMS-based approaches have also been shown to dramatically improve the identification of peptide subsets including cross-linked peptides (46), cysteine-containing peptides (47), and glycopeptides (48). Inspired by these previous studies, we set out to assess whether FAIMS-based analysis would allow for deep proteomic coverage and simultaneous assessment of the N-terminome on limited samples, yielding a cheaper and more streamlined method to identify protease substrates. We used the protease legumain as a model system.

Legumain is a cysteine protease with unique preference to cleave substrates after asparagine residues (49). Following synthesis as an inactive zymogen, it is trafficked to the endo-lysosomal pathway *via* mannose-6-phosphate-dependent mechanisms. Upon reaching acidic environments, legumain cleaves itself to produce a mature, proteolytically active enzyme (50, 51). Legumain protease activity favors acidic conditions, and its active conformer is thought to be rapidly destroyed upon entering neutral environments (51). Increasing evidence suggests extra-lysosomal localization of legumain and that it can cleave substrates in these environments, including the nucleus (52), cytoplasm (53), and extracellular space (54). While binding to integrins *via* its RGD motif is postulated to stabilize active legumain at the cell surface, how it remains active in the nucleus and cytoplasm is not well understood.

Legumain contributes to renal homeostasis and lysosomal protein turnover, as evidenced by renal insufficiency and lysosomal storage disorders in legumain-deficient mice (55, 56, 57). Legumain activity is upregulated in a range of diseases, including Alzheimer’s and Parkinson’s diseases (58, 59, 60), pancreatitis (61, 62), and cancer (63, 64). Inhibiting legumain reduced synapse loss and cognitive impairment in tauopathy mice (65, 66) and reduced α-synuclein cleavage in SNCA-transgenic mice to improve dopamine levels and motor functions (67). In an MMTV-PyMT murine breast cancer model, blocking legumain activity decreased lung metastasis (63). These studies suggest that targeting legumain activity has strong therapeutic potential. The proteolytic events leading to these observed phenotypes, however, are yet to be fully elucidated. To date, relatively few legumain substrates have been identified (49), among which include the invariant chain (68, 69), pro-MMP-2 (70), endosomal toll-like receptors (71), and the nuclear protein FOXP3 (72). Recent studies have aimed to systematically identify legumain substrates by spiking recombinant legumain into acidified lysates (73, 74). In these *in vitro* conditions, legumain cleaves hundreds of proteins after asparagine residues and at lower pH, also after aspartate residues. Identification of physiological substrates, where cellular compartmentalization and pH environments are intact, however, is lacking. To better understand the proteolytic contribution of legumain to cellular function and disease, an unbiased and systematic approach to identify its native substrates is required.

In the current study, we benchmarked our FAIMS-facilitated N-terminomics method in mouse macrophages treated with the legumain inhibitor SD-134 (75), revealing significant improvements in the coverage of N-termini compared to unfractionated samples. We then analyzed naïve spleens from WT and legumain-deficient (*Lgmn*^*−/−*^) mice to reveal global alterations in proteolysis, including 119 putative legumain substrates. Our data provide the first comprehensive list of physiological legumain substrates which provides insight into novel functions of legumain in neutral cellular environments. FAIMS-facilitated N-terminomics is thus a streamlined and accessible method to identify novel proteolytic substrates.

## Experimental Procedures

### Cell Culture

RAW264.7 cells (mouse monocyte/macrophage) were cultured in Dulbecco’s Modified Eagle Medium (DMEM, high glucose, Thermo Fisher Scientific) supplemented with 10% fetal bovine serum (FBS, CellSera) and 1% antibiotics (100 U/ml penicillin-streptomycin, Thermo Fisher Scientific) at 37 °C with 5% CO_2_. Cells were passaged 1:10 once reaching 80 to 90% confluence using a cell scraper.

### Mice

*Lgmn*^−/−^ C57BL/6N were a gift from Thomas Reinheckel (76). Mice were bred in the laboratory of Brian Schmidt and Nigel Bunnett at New York University and studies were approved by the NYU Institutional Animal Care and Use Committee. Splenic tissues were harvested from 8-week-old healthy male mice (WT and *Lgmn*^*−/−*^), snap frozen, and stored at −80 °C.

### Inhibition of Legumain and Assessment of Legumain Activity

The legumain-specific inhibitor SD-134 (75) was used to inhibit legumain in RAW264.7 cells. Cells (2 × 10^6^) were seeded in 6-well plates, followed by the addition of vehicle or SD-134 (10 μM added from a 10 mM stock; 0.1% final DMSO concentration). After 16 h, cells were harvested and used for MS analysis as below. Alternatively, the activity-based probe LE28 (77) was used to assess residual legumain activity and inhibitor efficacy. Cells were lysed in citrate buffer (50 mM citrate (Thermo Fisher Scientific, pH 5.5), 0.5% CHAPS (Sigma), 0.1% Triton X-100, 4 mM DTT (Sigma)), and solids were cleared by centrifugation (21,000*g*, 5 min, 4 °C). Protein concentrations in the resulting supernatant were determined by bicinchoninic acid (BCA) assay according to manufacturer’s instructions (Pierce). Total protein (100 μg) was diluted in 20 μl citrate lysis buffer and LE28 (1 μM) was added from a stock of 100 μM (1% final DMSO concentration). After 30 min at 37 °C, the reaction was quenched by the addition of 5x sample buffer (50% glycerol (Sigma), 250 mM Tris–Cl (Sigma), pH 6.8, 10% SDS (VWR LifeSciences), 0.04% bromophenol blue (Sigma), 6.25% beta-mercaptoethanol (Sigma), diluted to 1x final). Samples were boiled at 95 °C for 5 min, and proteins were resolved on a homemade 15% SDS-PAGE gel. To detect LE28-labeled species, gels were scanned with a Cy5 filter set on a Typhoon flatbed laser scanner (GE Healthcare). Spleens were similarly analyzed with LE28 to confirm loss of activity in *Lgmn*^*−/−*^ tissue, except lysis in citrate buffer was facilitated by sonication.

### Immunoblotting

Proteins were transferred to nitrocellulose membranes using the *Trans*-Blot Turbo Transfer system (Bio-Rad) and incubated with the indicated primary antibody overnight at 4 °C. Blots were washed in PBS containing 0.1% Tween-20 (PBST; Sigma) three times before incubation with the secondary antibody for 1 h at room temperature and three washes with PBST. A final wash in PBS was performed prior to detection. Horseradish peroxidase–conjugated antibodies were detected with Clarity ECL Substrate (Bio-Rad) on a ChemiDoc (Bio-Rad). Fluorophore-conjugated antibodies were visualized using the Typhoon 5 IRlong channel. Ponceau S stain was used to evaluate loading and transfer efficacy. All antibodies were diluted in 1:1 Intercept blocking buffer (LI-COR) and PBST. Bands were quantified by densitometry using ImageJ (Fiji) with background subtraction. Antibodies used in this study included goat anti-mouse legumain (1:1,000, R&D AF2058), goat anti-mouse elastase 2A (1:1,000, R&D AF4517), rabbit anti-β-actin (1:10,000, Life Technologies, MA5-15739), donkey anti-goat IgG HRP-conjugated (1/10,000, Novex Life Technologies, A15999), goat anti-rabbit IgG IR800-conjugated (1:10,000, Li-cor, 926-32213).

### *In Vitro* Recombinant Protein Cleavage Assay

To assess the direct cleavage of various proteins by legumain *in vitro*, we incubated recombinant proteins (Supplemental Table S33) with activated recombinant human legumain (1.5 μg/μl stock, gifted by Hans Brandstetter, 0.015 μg/μl final concentration) at 1:1 mass ratio (0.3 μg each) in acetate buffer (50 mM sodium acetate (ChemSupply), 100 mM sodium chloride (EMSURE), pH 5.5) and incubated at 37 °C for 3 or 5 h. Samples in the absence of legumain were used as a negative control. SD-134 (100 μM final concentration) was also pre-incubated with legumain for 2 min at 37 °C to inhibit legumain protease activity prior substrate addition, with DMSO used as a vehicle control. The reactions were quenched by the addition of 5x sample buffer (1x final concentration) prior to analysis by SDS-PAGE. Band visualization was achieved using 0.1% Coomassie brilliant blue G-250 dye (Bio-Rad) in 50% methanol, 10% acetic acid. Briefly, gels were stained in Coomassie solution for 30 min at room temperature with shaking followed by three rounds of destaining in 30% ethanol, 10% methanol for 10 min each. Gels were rinsed in MilliQ water overnight prior to imaging on the Typhoon 5 IRlong channel. Alternatively, following a 5-h incubation at 37 °C, samples were quenched with 4% SDS, 250 mM Tris–HCl (pH 6.8) and prepared for N-terminomic analysis.

### Protein N-termini Dimethylation and Proteome Preparation

RAW264.7 cells were treated with DMSO or 10 μM SD-134 as above and harvested (n = 4/group). Splenic tissue was harvested from WT C57BL/6 and *Lgmn*^*−/−*^ mice and stored at −80 °C (n = 4/group). Cells and tissues were lysed by sonication in 4% SDS, 50 mM Hepes (pH 7.5, Sigma) containing Roche cOmplete, EDTA-free protease inhibitor (Sigma). After boiling for 10 min, lysates were cleared by centrifugation (21,000*g*, 5 min, 4 °C) and total protein (100 μg) was diluted in 100 μl buffer according to BCA analysis. Recombinant proteins from the *in vitro* cleavage assay were prepared as described above.

Proteins were reduced with 20 mM DTT (80 °C, 10 min, 500 rpm) and alkylated with 50 mM iodoacetamide (37 °C, 30 min, 500 rpm) in the dark followed by quenching with 50 mM DTT (37 °C, 20 min, 500 rpm). Paramagnetic beads (Sera-Mag SpeedBeads 45152105050250 and 65152105050250, GE Healthcare) were prepared by mixing in a 1:1 ratio and washing three times in Milli-Q water before adjusting to a final concentration of 50 μg/μl in Milli-Q water as previously outlined (78). Conditioned paramagnetic SP3 beads were added to samples (2 mg of SP3 beads, final protein:SP3 bead ratio of 1:20) and protein aggregation was initiated with the addition of ethanol (80% final concentration). Samples were then gently shaken (25 °C, 1000 rpm) for 20 min prior to washing three times with 500 μl of 80% ethanol using a magnetic rack and resuspending in 90 μl of 6 M guanidine hydrochloride, 100 mM Hepes (pH 7.5). Proteins were dimethylated by adding 30 mM formaldehyde (Sigma) and 30 mM sodium cyanoborohydride (Sigma) and shaking (37 °C, 1000 rpm) for 1 h. This was repeated once more with an additional 30 mM of formaldehyde and 30 mM sodium cyanoborohydride before labeling was quenched by adding 25 μl 4 M Tris-base (pH 6.8) and shaking (37 °C, 1000 rpm) for 1 h. Excess formaldehyde and sodium cyanoborohydride were removed from samples using SP3 clean up (1 mg SP3 beads; final protein:SP3 bead ratio of 1:30) and proteins were precipitated with ethanol (80% final concentration). Samples were gently shaken (25 °C, 1000 rpm) for 20 min and then washed three times with 500 μl of 80% ethanol using a magnetic rack. SP3 beads were then resuspended in 100 μl of 200 mM Hepes (pH 7.5) and digested overnight at 37 °C with Solu-trypsin (3 μg solu-trypsin, Sigma, trypsin:protein ratio 1:33). The resulting peptide mixtures were collected using a magnetic rack, acidified with buffer A∗ (0.1% TFA, 2% acetonitrile) and desalted using C18 StageTips (Empore, 3M) with the addition of Oligo-R3 resin reverse phase material (Thermo Fisher Scientific) as previously described (79, 80). Samples were dried using a speedvac and stored at −20 °C until analysis.

### Online Fractionation by High-Field Asymmetric Waveform Ion Mobility Spectrometry and Mass Spectrometry Analysis

Proteome samples were resuspended in buffer A∗ and separated using a two-column chromatography setup composed of a PepMap100 C_18_ 20-mm by 75-μm trap and a PepMap C_18_ 500-mm by 75-μm analytical column (Thermo Fisher Scientific) on a Dionex Ultimate 3000 UPLC (Thermo Fisher Scientific). Samples were concentrated onto the trap column at 5 μl/min for 5 min with buffer A (0.1% formic acid, 2% DMSO) and then infused into an Orbitrap 480 mass spectrometer (Thermo Fisher Scientific) equipped with a FAIMS Pro interface at 300 nl/min. For each sample/FAIMS fraction ∼2 μg of peptide mixtures was separated using 125-min analytical runs undertaken by altering the buffer composition from 3% buffer B (0.1% formic acid, 77.9% acetonitrile, 2% DMSO) to 23% B over 95 min, then from 23% B to 40% B over 10 min, then from 40% B to 80% B over 5 min. The composition was held at 80% B for 5 min, and then dropped to 2% B over 0.1 min before being held at 2% B for another 9.9 min. For each sample, six individual LC-MS runs were collected with the Orbitrap 480 Mass Spectrometer operated using different FAIMS compensational voltages (CV) of either −35, −45, −55, −65, −75 or −85. For each FAIMS fraction, data-dependent acquisition was undertaken with a single Orbitrap MS scan (300–2000 m/z, a resolution of 120k with the Automated Gain Control (AGC) set to a maximum of 300%) collected every 3 s followed by Orbitrap MS/MS HCD scans of precursors (Normalized collision energy of 30%, maximal injection time of 50 ms, a resolution of 30k and a AGC of 250%). Non-FAIMS analysis was undertaken using the same LC-MS/MS parameters as outlined above on the same biological samples used for FAIMS analysis.

Dimethylated and trypsin digested *in vitro* cleavage assay samples were re-suspended in buffer A∗ and separated on a two-column chromatography setup composed of a PepMap100 C_18_ 20-mm by 75-μm trap and a PepMap C_18_ 500-mm by 75-μm analytical column (Thermo Fisher Scientific) on a Dionex Ultimate 3000 UPLC (Thermo Fisher Scientific) coupled to a Q Exactive Plus Orbitrap mass spectrometer (Thermo Fisher Scientific). Each sample (3 μg) were concentrated onto the trap column at 5 μl/min for 5 min with buffer A and then infused into the mass spectrometer at 300 nl/min. Samples were separated using 65-min analytical runs undertaken by altering the buffer composition from 2% buffer B to 23% B over 35 min, then from 23% B to 40% B over 10 min, then from 40% B to 80% B over 5 min. The composition was held at 80% B for 5 min, and then dropped to 2% B over 0.1 min before being held at 2% B for another 9.9 min. Data-dependent acquisition was undertaken with a single Orbitrap MS scan (375–2000 m/z, a resolution of 70k with the Automated Gain Control (AGC) target set to 3 × 10^6^ and maximal injection time of 50 ms) followed by up to five HCD scans (Stepped HCD Normalized collision energy of 28%, 30%, and 32%, resolution of 17.5k with a AGC target set to 2 × 10^5^ and maximal injection time of 100 ms) and parallel reaction monitoring (PRM (81)) of peptides of interest (Stepped HCD Normalized collision energy of 30%, 35%, and 40%, resolution of 35k with an AGC target set to 2 × 10^5^ and maximal injection time of 110 ms). The PRM *m/zs* of peptides of interest were based on the double, triple, or quadruple-charged states of the human protein sequences (Supplemental Table S34).

### Quantitative Proteomics and N-terminomics Data Analysis

RAW264.7 cell lysate and murine spleen lysate data files were processed and searched using MSFragger (Fragpipe v.18.0) (82) against the unreviewed murine proteome (*Mus musculus*, UniProt Accession: UP000000589, downloaded June 2022, 17,230 protein entries), supplemented with common contaminants, and a reverse decoy database (17,230 decoys: 50%). All six FAIMS fractions for a given sample were defined as a single biological replicate with individual FAIMS CVs defined as fractions and experiments searched all together to ensure a global false discovery rate fetal bovine serum (FDR) of 1% (83). Parameters were set to default unless otherwise described below. Identification and label-free quantification (LFQ) were undertaken allowing for cysteine carbamidomethylation as a fixed modification (+57.0215 Da) as well as variable modifications of lysine dimethylation (+28.0313 Da), methionine oxidation (+15.9949 Da), N-terminal acetylation (+42.0106 Da), N-terminal cyclization (−17.0265/−18.0106 Da), N-terminal dimethylation (+28.0313 Da), and N-terminal lysine dimethylation (+56.0626 Da). Cleavage specificity was set to “SEMI-N_TERM” and “TrypsinR” (Arg-C), allowing a maximum of 2 missed cleavages. Precursor and fragment mass tolerances of 20 ppm and isotopic error of 3 Da were also included. Protein and peptide-level FDR were determined using Philosopher (v.4.3.0) with default settings (FDR threshold set at 1%). Quantification parameters were left as default and performed with IonQuant (v.1.8.0) (84). The resulting outputs (MaxLFQ values) were further processed in Perseus (v.1.6.0.7) (85), removing reverse decoy matches before a log_2_ transformation was applied. Protein/peptides identified in a minimum of three of four biological replicates in at least one of the groups (DMSO/SD-134 or WT/*Lgmn*^*−/−*^) were selected and missing values imputed based on a downshifted normal distribution (σ-width = 0.3, σ-downshift = −1.8) for statistical analyses at the protein and N-termini level. Due to legumain cleavage events being absent in *Lgmn*^*−/−*^ samples, imputation was used to allow statistical analysis to guide the identification of cleavage events overrepresented within WT samples. Student’s two-sample *t* test was applied for statistical comparison between groups with a significance threshold set to log_2_(fold change) ±1 and -log_10_(p) = 1.3 (*p* = 0.05). Volcano plots, charts, heatmaps, principal component analyses, upset plots, and Venn diagrams were all created using R (v.4.2.0). Enrichment analyses using Fisher exact tests were undertaken in Perseus and visualization of proteomic data undertaken in the R statistical environment using the ggplot2 package (v.3.3.6) (86). Pearson correlation and statistical summary analyses were performed in Perseus and standard deviations taken for visualization. The identification of C-terminal peptides was achieved according to the approach of Bell *et al.* (37) using MSFragger (Fragpipe v.20.0) with identical parameters as above, except with the cleavage specificity altered to “SEMI” to detect peptides with non-tryptic C-terminal ends. C-termini were considered to be true C-terminal peptides if they possessed Arg-C specificity at the N-terminus but not at their C-terminus.

Recombinant protein cleavage assay data files were searched against a custom human database containing sequences for legumain (Uniprot accession: Q99538), cathepsin S (Uniprot accession: P25774), lysosomal α-mannosidase (Uniprot accession: O00754), lamina-associated polypeptide 2 (Uniprot accession: P42167), and tyrosyl-tRNA synthetase (Uniprot accession: P54577) with common contaminants and a reverse decoy database added by MSFragger (Fragpipe v.18.0) (242 entries including 121 decoys). Identification and quantification of peptides occurred as described above. *In vitro* cleavage assay spectra were manually assessed and annotated with the Interactive Peptide Spectral Annotator (87).

### Bioinformatic Analysis of Protein and Peptide Data

Data were processed in WebPICS (88) and TopFINDer (89, 90) for generation of sequence logos using plogo (91). STRING-dp (v.11.5) was used for protein interaction and pathway analyses (https://string-db.org) with medium confidence (0.400) and FDR stringency (5%). Subcellular localization analysis was undertaken based on GO terms associated with Uniprot accession UP000000589.

### MPO Activity Assay

Splenic tissues were sonicated in 50 mM potassium phosphate buffer (pH 6.0) containing 0.5% hexadecyltrimethylammonium bromide (Sigma) using the method described above. Total protein was normalized by BCA (7 μg in 7 μl lysis buffer) and aliquoted into a Corning Costar 96-well flat bottom clear plat. Potassium phosphate buffer (50 mM, pH 6.0) containing 0.167 mg/ml O-dianisidine-HCl (Sigma), 0.0005% H_2_O_2_ (Sigma) was added, and absorbance was read at 460 nm every 30 s for 30 min on the Clariostar Omega Plate Reader (BMG Labtech). Linear values were taken to calculate slopes and graph results.

### Experimental Design, Statistical Rationale, and Data Availability

For FAIMS-facilitated N-terminomics analysis of RAW264.7 cells, a total of four replicates per treatment (DMSO or SD-134, n = 4) were included for LC-MS/MS analysis to ensure sufficient statistical power in subsequent analyses. For statistical testing in Perseus (v.1.6.0.7), peptide-spectrum matches were only included if identified in at least 3 replicates in one of the groups, where a student’s two-sample *t* test was applied to compare sample means, assuming random sampling from independent groups of normal populations. For naïve murine spleen tissue, four biological replicates per group (WT and *Lgmn*^*−/−*^, n = 4) were used for N-terminomics analysis to ensure representation of the population. Statistical analysis was performed as described above. Recombinant protein cleavage assays were performed with four replicates per group (−/+ LGMN, n = 4) to ensure reproducibility and robust statistical analyses. For immunoblots and the myeloperoxidase activity assay, five biological replicates per group (WT and *Lgmn*^*−/−*^, n = 5) were used to ensure reproducibility and statistical power. Statistical analyses were performed using GraphPad Prism 9 unless otherwise stated with all data presented as mean ± SEM and significance set at *p* < 0.05. All pairwise comparisons were analyzed using a student’s *t* test assuming normal distributions.

## Results

### FAIMS Fractionation Enables Deep Proteome Coverage and Identification of Native Cleavage Events

**Fig. 1:**
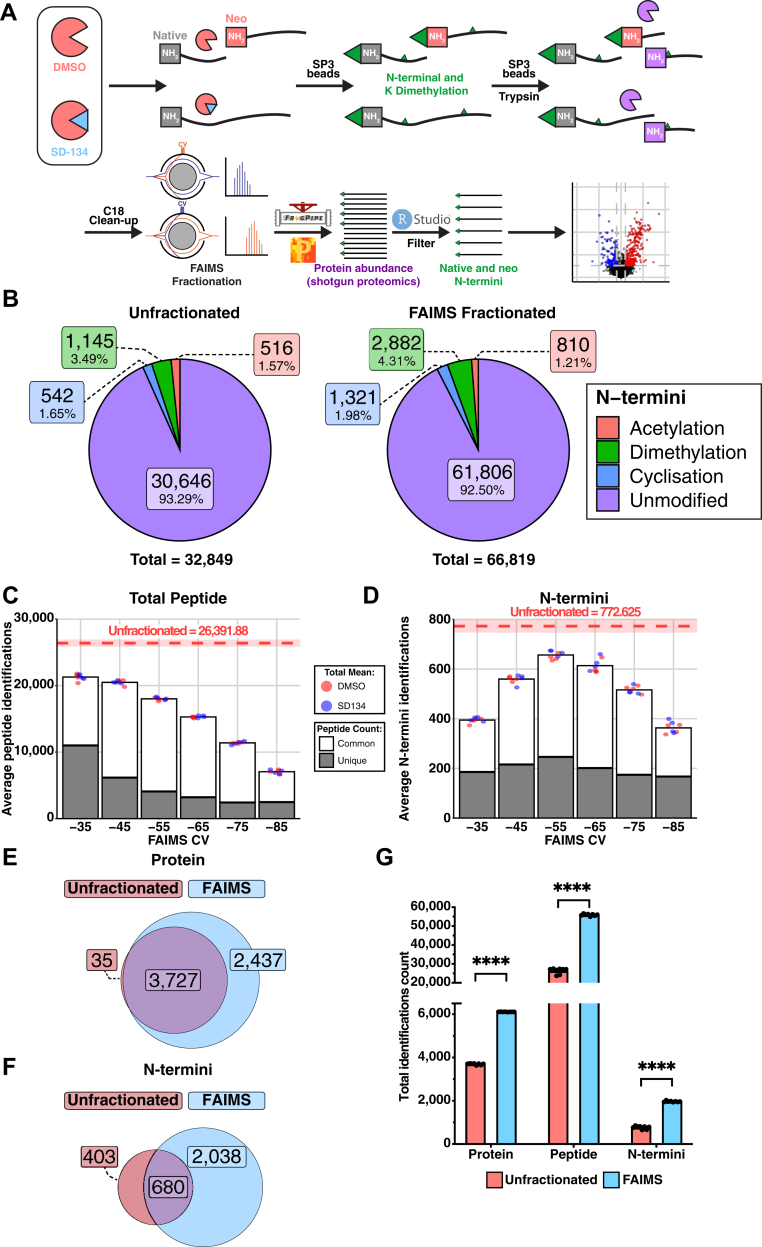
**FAIMS-facilitated N-terminomics increases overall peptide detection compared to unfractionated methods.***A*, experimental workflow. RAW264.7 cells treated with DMSO (n = 4) or 10 μM SD-134 (n = 4) and naïve spleen tissue from WT (n = 4) and legumain-deficient (*Lgmn*^*−/−*^, n = 4) mice were analyzed by FAIMS-facilitated N-terminomics. Native and neo-N-termini were labeled with formaldehyde and peptides digested by trypsin. Online gas-phase fractionation was achieved using FAIMS (high-field asymmetric wavefield ion mobility spectrometry) over a range of compensation voltages (CV, −35, −45, −55, −65, −75, −85) prior to mass spectrometry analysis. Data were analyzed by MSFragger (Fragpipe) and Perseus. Native cleavage sites were bioinformatically enriched by filtering for N-terminal dimethylation using RStudio. Numbers shown refer to peptide-spectrum matches present in at least three of four biological replicates in a minimum of one group (*B*–*F*). *B*, peptide-spectrum matches and their N-terminal modifications identified in unfractionated (*left panel*) and FAIMS-fractionated (*right panel*) RAW264.7 cell lysates. *C* and *D*, average total peptides (*C*) and dimethylated N-terminal peptides (neo-N-termini) (*D*) identified in each biological replicate per FAIMS fraction. *Red dashed line* refers to average peptides or N-termini identified without FAIMS fractionation with SD indicated by the box. Peptide-spectrum matches identified in only one specified CV fraction are indicated in *gray* (unique to that fraction) and those identified in multiple CV fractions are shown in *white* (common between fractions). *E* and *F*, Unique protein (*E*) and N-termini (*F*) identifications and their overlap (*purple*) between unfractionated (*red*) and FAIMS-fractionated (*blue*) RAW264.7 cell lysates. *G*, average number of proteins, peptides, and N-termini identified in each biological replicate from unfractionated (*red*) and FAIMS-fractionated (*blue*) RAW264.7 cell lysates. A student’s *t* test was performed for pairwise comparisons. (∗∗∗∗*p* < 0.0001). CV, compensation voltage.

Conventional N-terminomics techniques often rely on selection methods to enrich N-terminal peptides (19). While effective, enrichment is performed at the cost of bulk proteome data, limiting the ability to assess if observed alterations are true changes in the N-termini or the global proteome. To overcome this, we assessed the potential to undertake simultaneous proteome and N-terminome analyses by coupling FAIMS fractionation to established dimethylation-based N-termini labeling (27, 32). Leveraging fragment-ion indexing-based proteomic searches using MSFragger (82), we explored the ability to identify N-termini sequences modified with acetylation, N-terminal cyclization events (pyro-Glu and pyro-Gln), as well as N-terminal dimethylation modification events on semi-tryptic peptides (Fig. 1*A*). We reasoned this would enable identification and quantitation of both protein abundance and cleavage event (neo-N-termini) information from limited amounts of complex samples such as tissue.

We applied FAIMS to analyze the global proteome and N-terminome of RAW264.7 murine macrophages in response to the legumain-specific inhibitor SD-134 (75). Using the fluorescently quenched activity-based probe for legumain, LE28, we confirmed the ability of SD-134 to inhibit legumain (77) (Supplemental Fig. S1). To investigate whether FAIMS could improve N-terminome analysis, protein lysates were dimethylated, digested with trypsin and subjected to online gas-phase fractionation using six FAIMS compensation voltages (CVs: −35, −45, −55, −65, −75, and −85) (Supplemental Tables S1, S2, S6 and S7), with each analytical run utilizing 2 μg digested material. This was benchmarked against identical analysis in the absence of FAIMS fractionation. A total of 32,849 unique peptides corresponding to 3762 proteins were identified in unfractionated samples, while FAIMS permitted detection of 66,819 peptides corresponding to 6164 proteins (2.03-fold increase) (Fig. 1*B*, Supplemental Tables S3, S4, S8 and S9). The dimethylation labeling efficacy was observed to be >95% (Supplemental Fig. S2). Fractionation of samples by FAIMS provided access to a greater number of N-termini (2,882, 4.31% of total peptides) than unfractionated samples (1,145, 3.49% of total peptides) (Fig. 1*B*; Supplemental Tables S5, S10 and S11). Furthermore, as each FAIMS fraction yielded a substantial number of peptide and N-termini identifications exclusive to that CV value (Fig. 1, *C* and *D*), with increasing percentage of N-termini identified as the CV goes from −35V to −85V (Supplemental Fig. S3), coverage of the N-terminome was improved.

While the majority of proteins and N-termini identified within the unfractionated samples were also identified after FAIMS fractionation, the improved proteome depth of this approach lead to detection of an additional 2038 unique N-termini (Fig. 1, *E* and *F*). The identification of 403 N-termini unique to the unfractionated samples also highlights an important nuance associated with FAIMS analysis, which is that not all peptides identified under unfractionated conditions will be identified within a given FAIMS CV or even across multiple FAIMS CVs, as highlighted previously (92, 93). Of the quantified N-termini, approximately 95% had complete protein quantifications in at least one of the biological groups, which was marginally elevated compared to unfractionated samples (90%) (Supplemental Fig. S4). Overall, FAIMS fractionation identified significantly more proteins, peptides, and N-termini per biological replicate compared to unfractionated samples (Fig. 1*G*). We observed tighter distributions and significant reductions in the standard deviation and coefficient of variation for protein and N-termini quantifications following FAIMS fractionation, demonstrating that the quality of the data was also improved (Supplemental Fig. S5, *A*–*D*). When comparing our results to our previously published TAILS dataset obtained from RAW264.7 cells treated with the legumain inhibitor LI-1 (94) (Supplemental Fig. S6), we detected a similar number of N-termini in the two methods, despite using ∼10x less starting material than in the TAILS analysis. Hence, FAIMS-facilitated N-terminomics circumvents the requirement of N-termini enrichment, allowing assessment of both the proteome and N-terminome using limited sample amounts.

**Fig. 2:**
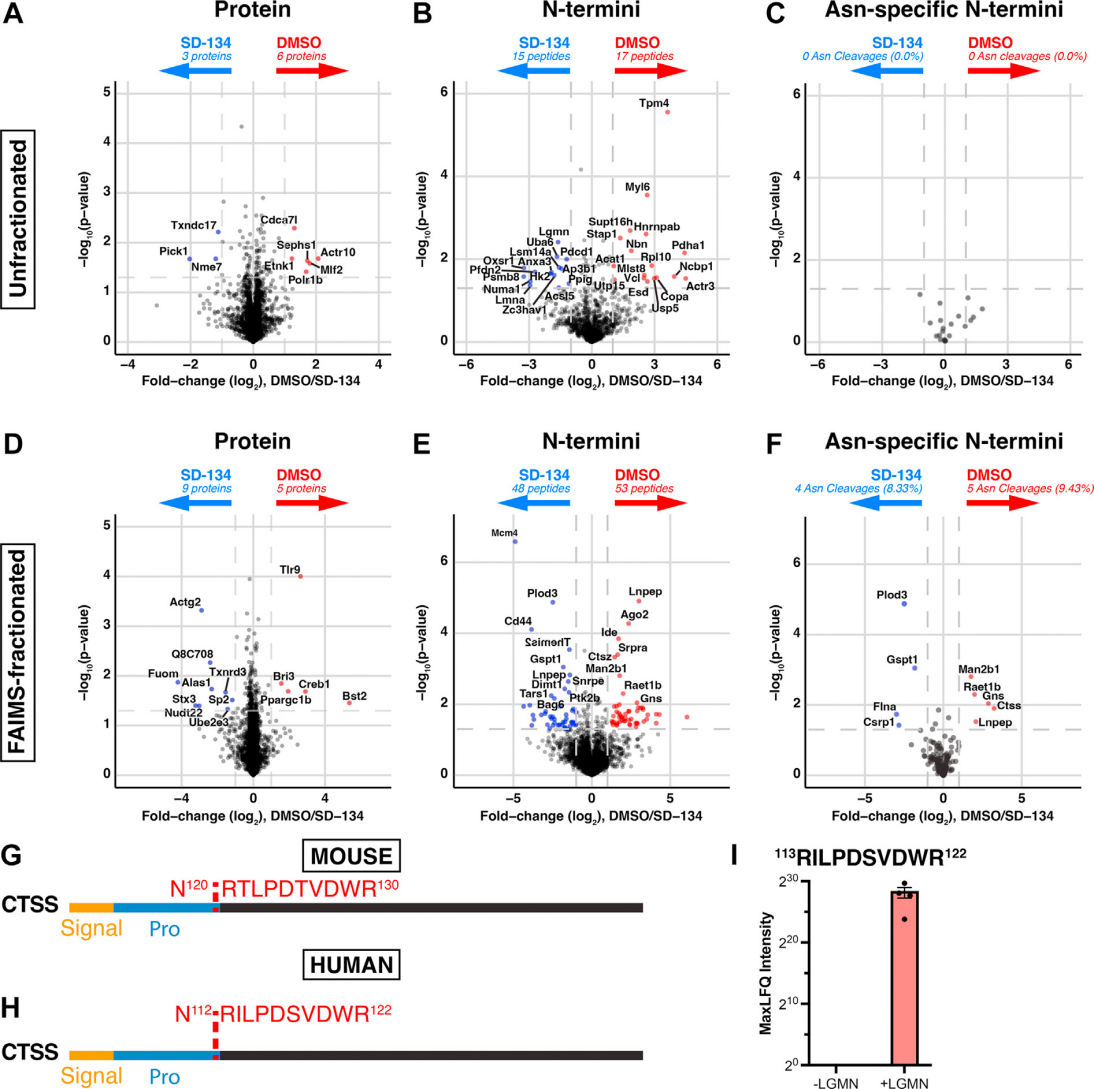
**Quantitative proteomics and N-terminomics analyses of unfractionated and FAIMS-fractionated DMSO and SD-134–treated RAW 264.7 cell lysates.** Peptide-spectrum matches were analyzed using Perseus, and proteins were required to have valid values in at least three of four biological replicates in at least one group to be considered for further analysis. *A*–*F*, for unfractionated (*A*–*C*) and FAIMS-fractionated (*D*–*F*) data, protein (*A* and *D*) and N-termini (*B* and *E*) identifications were analyzed by a two-way *t* test and visualized by volcano plot where significance is defined as abs(log_2_(DMSO/SD-134)) > 1 and -log10(p) > 1.3. Neo-N-termini arising from cleavage after asparagine residues were also identified (*C* and *F*). *G*, schematic of murine cathepsin S (CTSS) cleavage at N^120^ ↓ R^121^ as identified by FAIMS-facilitated N-terminomics. Cleavage site is shown in *red*, signal peptide in *orange*, and propeptide in *blue*. *H*, schematic representation of human cathepsin S cleavage by legumain at N^112^ ↓ R^113^ as identified by N-terminomics analysis of recombinant protein cleavage assay. Identified cleavage site is shown in *red*, signal peptide in *orange*, and propeptide in *blue*. *I*, MaxLFQ intensities for the neo-N-terminus ^113^RILPDSVDWR^122^ as identified by N-terminomics analysis of recombinant protein cleavage assay with cathepsin S (CTSS) and in the presence (+) or absence (−) of legumain (LGMN).

Compared to unfractionated analysis, FAIMS fractionation also produced an expanded set of proteins and N-termini exhibiting significant differences between DMSO- and SD-134-treated samples (Fig. 2, *A*, *B*, *D* and *E*; Supplemental Tables S3, S5, S8 and S10). We hypothesize that missing data in the unfractionated analysis was due to the reduced proteome coverage (Fig. 1*B*) compared to the FAIMS analysis. Nevertheless, protein and N-termini quantification is highly correlative between the unfractionated and FAIMS-fractionated data (Supplemental Fig. S7). To identify legumain-specific cleavage events, we filtered the dimethylated N-termini for those that arose due to cleavage after asparagine residues. While no asparaginyl cleavages were significantly enriched in unfractionated DMSO-treated samples (Fig. 2*C*; Supplemental Table S5), five were detected by the FAIMS method (Fig. 2*F*; Supplemental Tables S10 and S11). One of these cleavage sites was within another lysosomal protease, cathepsin S; the neo-N-terminus identified corresponded to cleavage at N^120^↓ R^121^, immediately upstream of the canonical pro-cathepsin S cleavage site (Fig. 2*G*). Using unfractionated N-terminomics analysis, we demonstrated that legumain cleaves human cathepsin S *in vitro* at the conserved site N^112^ ↓ R^113^ (Fig. 2, *H* and I, Supplemental Fig. S8; Supplemental Table S12). Together, our data demonstrate that FAIMS-facilitated N-terminomics is an effective workflow for the sensitive detection of N-termini from complex samples, allowing protease substrate identification without enrichment.

### FAIMS-Facilitated Analysis of Legumain-Deficient Mouse Spleens Reveals Altered Proteolysis and Neutrophil Function

**Fig. 3:**
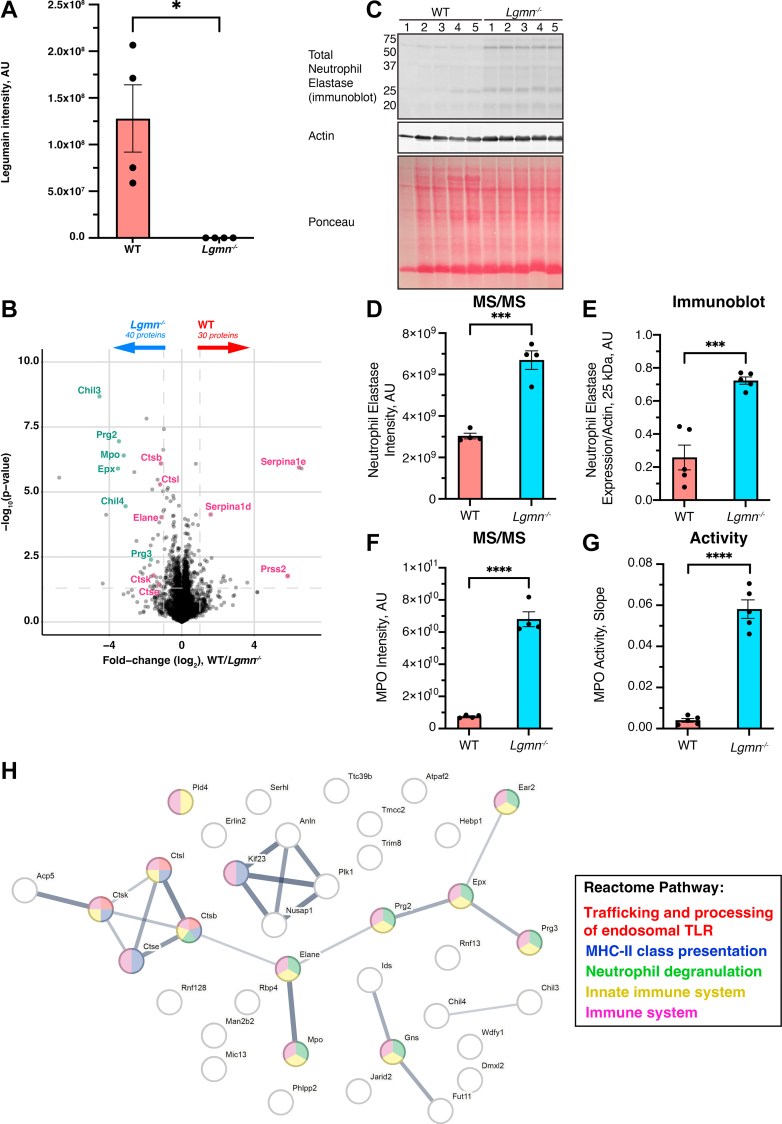
**Quantitative proteomics analysis of WT and legumain knockout (*Lgmn***^***−/−***^**) naïve mouse spleens by FAIMS-facilitated N-terminomics.***A*, legumain intensity values from quantitative proteomics analysis of WT and *Lgmn*^*−/−*^ naïve mouse spleens. Biological replicates are shown. A student’s *t* test was performed for pairwise comparisons (∗*p* < 0.05). Proteins identified in ≥3 of 4 biological replicates in at least one group (n = 4/group) were analyzed by a two-way *t* test and visualized by volcano plot (*B*). Significantly elevated proteins are defined by abs(log_2_(WT/*Lgmn*^*−/−*^)) > 1 and -log10(p) > 1.3. Altered proteins related to proteolysis are shown in *magenta* and those corresponding to neutrophils in *green*. *C*, total neutrophil elastase expression in naïve mouse spleen lysates measured by immunoblot. Actin and Ponceau S stain were used as loading controls. *D*, neutrophil elastase intensity measurements from proteomic analysis of WT and *Lgmn*^*−/−*^ naïve mouse spleens (n = 4/group). *E*, immunoblot bands were quantified by densitometry and normalized to actin (n = 5/group). *F*, myeloperoxidase intensity measurements from proteomic analysis of WT and *Lgmn*^*−/−*^ naïve mouse spleens (n = 4/group). *G*, mouse spleen lysates were analyzed by a myeloperoxidase activity assay and linear slope was calculated from 0 to 6 min (n = 5/group). A student’s *t* test was performed for pairwise comparisons (ns = not significant, ∗*p* < 0.05, ∗∗*p* < 0.01, ∗∗∗*p* < 0.001). *H*, STRING-db (v.11.5) analysis of the 40 *Lgmn*^*−/−*^ enriched proteins (confidence = 0.400, false discovery rate = 5%). Line thickness corresponds to the confidence of interaction. Reactome pathway: *red* = trafficking and processing of endosomal TLR, *blue* = MHC-II class presentation, *green* = neutrophil degranulation, *yellow* = innate immune system, *magenta* = immune system.

We next aimed to apply FAIMS-facilitated N-terminomics to examine the influence of legumain on the global proteome and N-terminome in a more physiologically relevant setting. As relatively little information is available on the role of legumain in spleen, we analyzed naïve spleens from WT and legumain-deficient (*Lgmn*^*−/−*^) mice (Supplemental Tables S13 and S14). We confirmed legumain was present and active in WT splenic lysates and absent in *Lgmn*^*−/−*^ lysates using the LE28 activity-based probe (77) and immunoblot (Supplemental Fig. S9); this was further verified in our LC-MS/MS analysis (Fig. 3*A*). We identified 64,649 peptides from 6366 proteins in our FAIMS-fractionated spleen lysates across all biological replicates (Supplemental Tables S15 and S16), where samples demonstrated clear clustering based on biological groups (Supplemental Fig. S10). Among the detected peptides, 2528 were dimethylated, with a labeling efficacy >95% (Supplemental Table S17; Supplemental Fig. S11). We analyzed various peptide properties between N-termini and all remaining peptides and observed no significant variations between the two groups (Supplemental Fig. S12). Additionally, each FAIMS fraction revealed a unique set of N-termini enabling deep N-terminome coverage (Supplemental Fig. S13).

We observed 30 proteins that were increased in abundance in the presence of legumain, including trypsin-2 (Prss2) and serine protease inhibitors (Serpina1e and Serpina1d) (Fig. 3*B*). Conversely, 40 proteins exhibited increased abundance in the absence of legumain including cathepsin B, L, E, and K, and several neutrophil-associated proteins (neutrophil elastase, myeloperoxidase, eosinophil peroxidase, proteoglycans, and chitinase-like proteins) (Fig. 3*B*, Supplemental Fig. S14). We verified an increase in neutrophil elastase in *Lgmn*^*−/−*^ spleens by immunoblot (Fig. 3, *C*–*E*). Likewise, myeloperoxidase activity was dramatically amplified in *Lgmn*^*−/−*^ spleens (Fig. 3, *F* and G). STRING (v.11.5) analysis of the 40 *Lgmn*^*−/−*^enriched proteins further revealed alterations in immune-related pathways including toll-like receptor (TLR) processing, MHC-II class presentation, and neutrophil degranulation (Fig. 3*H*; Supplemental Table S18). Consistently, proteins contributing to these pathways were overexpressed in *Lgmn*^*−/−*^ spleen lysates compared to WT counterparts (Supplemental Fig. S15). Overall, our proteomics data suggest the involvement of legumain in proteolytic regulation and neutrophil function.

### Identification of Novel Legumain Substrates in Naïve Mouse Spleens

**Fig. 4:**
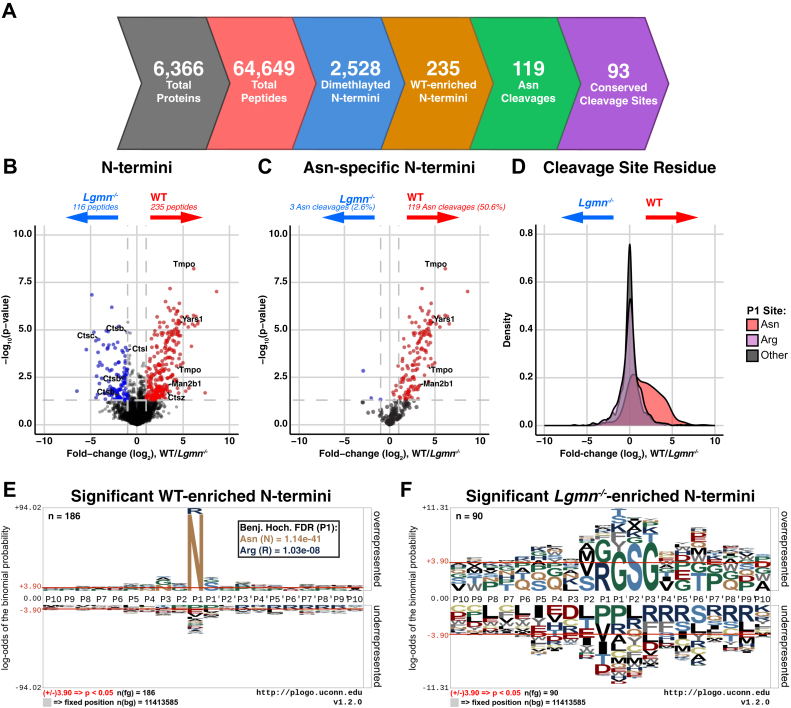
**FAIMS-facilitated N-terminomics analysis of WT and legumain-deficient (*Lgmn***^***−/−***^**) naïve mouse spleens.** Identified peptides were required to be present in at least three of four biological replicates in at least one group to be considered for analysis (n = 4/group). *A*, proteins and peptides identified in FAIMS-fractionated naïve mouse spleen lysates are summarized. Total peptide-spectrum matches were bioinformatically filtered for N-terminal dimethylation indicating endogenous N-termini. N-termini were further filtered for those arising due to cleavage after asparagine residues and for those that are conserved in both mouse and human proteins. A two-sample *t* test was performed and N-termini were visualized by volcano plot (*B* and *C*). *B*, WT-enriched N-termini are shown in *red* (log_2_(WT/*Lgmn*^*−/−*^) > 1 and -log_10_(p) > 1.3) and *Lgmn*^*−/−*^ in *blue* (log_2_(WT/*Lgmn*^*−/−*^) < −1 and -log_10_(p) > 1.3). *C*, asparaginyl cleavage events were also identified. *D*, density plot of data shown in (*B*) showing log_2_(WT/*Lgmn*^*−/−*^) distribution of N-termini. Peptides arising due to cleavage after asparagine residues are shown in *red*, arginine residues in *purple*, and all other residues in *gray*. *E* and *F*, sequence motifs of N-termini significantly enriched in WT (n = 186) (*E*) and *Lgmn*^*−/−*^ (n = 90) (*F*) naïve spleen lysates were created using plogo (O’Shea *et al.* 2013). Overrepresented amino acids appear above and underrepresented below the x-axis (*p* < 0.05).

To identify legumain-mediated alterations of the N-terminome, we compared the dimethylated N-termini observed between WT and *Lgmn*^*−/−*^ naïve mouse spleens. To quantify differences in N-termini, we imputed missing data (Supplemental Fig. S16) with the widely-used random drawing from a left-censored normal distribution approach, which is a conservative yet computationally straight forward approach for imputation (95). Of 2528 N-termini (Fig. 4*A*, Supplemental Table S17), 1443 of these were not within the first 65 amino acids of the proteins, consistent with endopeptidase activity (Supplemental Fig. S17, *A*–*C*). Statistical analysis (log_2_(fold change) > ±1 and -log_10_(p) > 1.3) revealed 235 cleavage events enriched in WT and 116 in *Lgmn*^*−/−*^ spleens (Fig. 4*B*, Supplemental Table S17). Amino acids flanking the observed cleavage sites were assessed to examine cleavage motifs enriched in WT and *Lgmn*^*−/−*^ tissue (Fig. 4, *E* and *F*). In line with the well-established asparaginyl endopeptidase activity of legumain, WT samples exhibited a strong enrichment in N-termini arising from cleavage after asparagine (Benj. Hoch. FDR = 1.14e^−41^, Supplemental Tables S19 and S20). In fact, 50.6% (119/235) of the WT-enriched N-termini were cleaved after Asn, while this was observed in only 2.6% (3/116) of the N-termini from *Lgmn*^*−/−*^ spleens (Fig. 4, *C* and *D*; Supplemental Tables S20 and S21).

Among the WT-enriched P1 asparaginyl cleavages (Supplemental Fig. S17*D*; Supplemental Table S22), we observed slight preferences for serine in the P1′ position, proline in the P3′, and glycine in the P2 and P3 positions. We also visualized the consensus motif for the non-asparaginyl cleavages (Supplemental Fig. S17*E*; Supplemental Table S23). Despite the well-established ability of legumain to cleave after aspartate residues in acidic pH, P1 Asp was only observed in 4 of the 116 non-asparaginyl cleavages in WT spleen. Notably, asparagine in the P3 position was enriched among these neo-N-termini (21/116). Of the 235 WT-enriched N-termini, the uncleaved peptides of 128 N-termini were also identified. These were either enriched in the *Lgmn*^*−/−*^ tissue or unchanged, further supporting legumain-dependent processing (Supplemental Fig. S17*F*). Six of 119 putative legumain cleavage events were also identified by Vidmar and colleagues using direct in-gel profiling of protease specificity (73); these included CGGBP1, EIF4G2, MAP2K1, HNRNPU, and PPM1G.

Inspired by the recent work of Bell and colleagues (37), we also searched our dataset with the cleavage specificity set to “SEMI,” which allows identification of peptides with non-tryptic C-terminal ends. We identified 110 C-termini that were enriched in WT spleen, and 287 enriched in *Lgmn*^*−/−*^ tissue (Supplemental Table S24, Supplemental Fig. S18*A*). Among the WT-enriched C-termini, 45 (40.91%) ended in asparagine residues, suggesting that they may have been generated through direct cleavage by legumain (Supplemental Figs. S18, *B* and *C* and S19*A*). Among the WT-enriched C termini that did not end in asparagine (P1), asparagine was notably observed in the P2′ position (Supplemental Fig. S19*C*).

Collectively, these data provide a comprehensive list of putative murine spleen legumain substrates in native conditions, shed light on cleavage preferences for legumain, and highlight divergent proteolysis in the absence of legumain.

### Putative Legumain Substrates Exhibit Extra-lysosomal Localization, Potentiating Legumain Proteolytic Activity in Neutral Environments

**Fig. 5:**
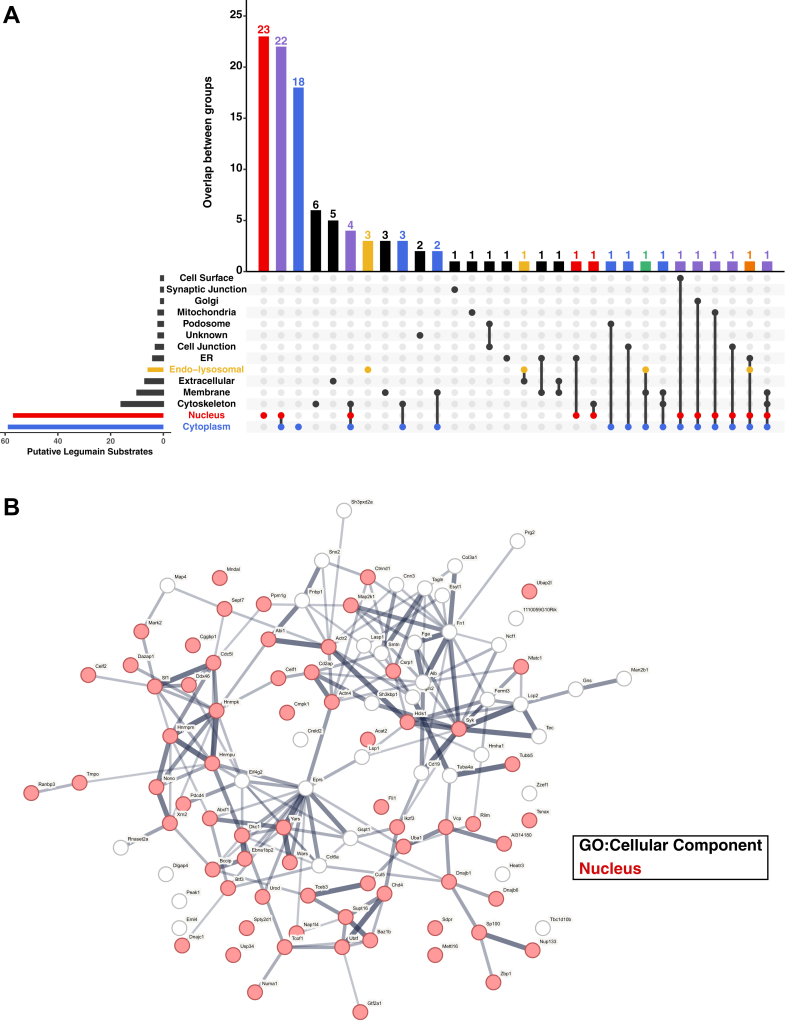
**Identification of putative legumain substrates in naïve mouse spleen lysates and characterization of their subcellular localization.** Legumain substrates were classified as neo-N-termini significantly enriched in WT naïve spleen lysates (log_2_(WT/*Lgmn*^*−/−*^) > 1 and -log_10_(p) > 1.3) containing an asparagine residue in the P1 site. *A*, upset plot of subcellular localizations of the 119 putative legumain substrates identified, as cataloged in Uniprot.org. Compartments of interest are highlighted such that *red* indicates nucleus, *blue* indicates cytoplasm, and *yellow* indicates endo-lysosomal system. Proteins localized to both nuclear and cytoplasmic regions are highlighted in *purple*, both cytoplasmic and endo-lysosomal in *green*, and all three nuclear, cytoplasmic, endo-lysosomal in *orange*. *B*, putative legumain substrates were further analyzed by STRING-db for gene ontology terms, cellular component (GO:CC). *Red* indicates nuclear localization of the protein.

We next investigated the 119 asparagine-specific neo-N-termini (corresponding to 110 proteins) that were enriched in WT spleens, which may result from direct cleavage by legumain (Fig. 4*C*). We plotted the 20 most differential neo-N-termini as a heatmap and observed clear reproducibility across biological replicates (Supplemental Fig. S17*G*). Ninety-three (78%) of the mouse cleavage sites exhibit an asparagine at the P1 position of the corresponding human proteins (Supplemental Table S25). When examining the localization of the putative substrates, only six are cataloged in UniProt as having endo-lysosomal localization (Fig. 5*A*; Supplemental Table S25). Instead, the majority (76%) are known to be localized to the nucleus and/or cytoplasm. Indeed, STRING (v.11.5) analysis of all observed putative legumain substrates indicated a high proportion with GO terms associated with the nucleus (GO:0005634, strength = 0.26, Benj. Hoch. FDR = 3.32e^−7^) (Fig. 5*B*; Supplemental Table S26). Analysis of the C-termini ending in asparagine (Supplemental Table S24) also indicated similar trends in the subcellular localization (Supplemental Fig. S18*D*).

**Fig. 6:**
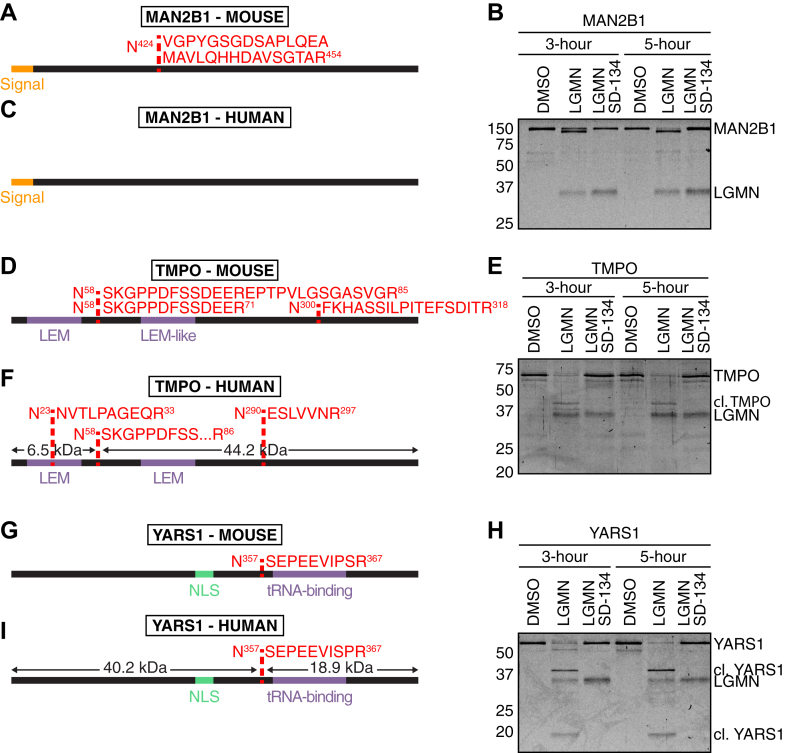
**Validating putative legumain substrates identified by FAIMS-facilitated N-terminomics.***A*, *D*, and *G*, schematic of murine lysosomal α-mannosidase (MAN2B1) cleavage at N^424^ ↓ V^425^ (*A*), lamina-associated polypeptide 2 (TMPO) cleavage at N^58^ ↓ S^59^ (*D*), and tyrosyl-tRNA synthetase 1 (YARS1) cleavage at N^357^ ↓ S^358^ (*G*) as identified by FAIMS-facilitated N-terminomics. Cleavage sites are shown in *red*, signal peptide in *orange*, key domains in *purple*, and nuclear localization signal (NLS) in *green*. *B*, *E*, and *H*, coomassie-stained 15% SDS-PAGE gels of recombinant human proteins incubated with activated recombinant legumain (LGMN) at pH 5.5 for 3 or 5 h (1:1 substrate: legumain mass ratio) in the presence or absence of 100 μM SD-134. DMSO was used as a vehicle control where samples were not incubated with either legumain or SD-134. *C*, *F*, and *I*, schematic representation of human lysosomal α-mannosidase (MAN2B1) (*C*), lamina-associated polypeptide 2 (TMPO) (*F*), and tyrosyl-tRNA synthetase 1 (YARS1) (*I*) asparaginyl cleavages as identified by N-terminomics analysis of recombinant protein cleavage assays. Dimethylated N-termini containing asparaginyl cleavages detected by LC-MS/MS analysis in legumain-treated samples are shown in *red*, signal peptide in *orange*, key domains in *purple*, and nuclear localization signal (NLS) in *green*. Predicted size of resulting cleavage products are shown. For the full dataset, see Supplemental Tables S27–S29.

We next aimed to validate three putative legumain substrates using an *in vitro* cleavage assay: lysosomal α-mannosidase (MAN2B1; cleaved at N^424^ ↓ V^425^), which was also identified in the RAW264.7 dataset, lamina-associated polypeptide 2 (TMPO; cleaved at N^58^ ↓ S^59^) and tyrosyl tRNA-synthetase 1 (YARS1; cleaved at N^357^ ↓ S^358^) (Table 1; Fig. 6, *A*, *D* and *G*). These putative substrates were selected as representatives from different subcellular localizations, including the lysosome, nucleus, and cytoplasm, respectively. We confirmed these substrates were not being differentially degraded *in vivo* as other detected tryptic peptides remained consistent between biological replicates, ensuring stability of the generated proteolytic products following legumain processing (Supplemental Fig. S20). We tested the ability of legumain to directly mediate these cleavages by incubating recombinant human proteins in the presence or absence of human legumain. Consistent with our *in vivo* identified cleavage events, legumain treatment resulted in notable alterations in gel mobility of the putative substrates TMPO and YARS1 (Fig. 6, *E* and *H*), however failed to replicate the expected MAN2B1 processing (Fig. 6*B* and Supplemental Fig. S21*A*). We hypothesize pre-processing of MAN2B1 may be required prior to cleavage by legumain and hence, we cannot detect cleavage *in vitro*. Addition of SD-134 confirmed that these proteolytic products were dependent on legumain activity (Fig. 6, *B*, *E* and *H*). We used dimethylation-based N-terminomics to identify these *in vitro* cleavage sites. These results support the cleavage of TMPO (N^58^ ↓ S^59^) (Fig. 6*F*, Supplemental Fig. S21*E*) and YARS1 (N^357^ ↓ S^358^) (Fig. 6*I*, Supplemental Fig. S21, *C* and *G*) at the residues observed *in vivo* (Supplemental Tables S27–S29). Additional asparaginyl cleavages were also observed for TMPO, suggesting potential degradation of TMPO by legumain *in vitro* (Supplemental Fig. S21, *B*, *D*–*F*). Overall, our data demonstrate that legumain possesses the capacity to process these recombinant proteins *in vitro* and validates the use of FAIMS-facilitated N-terminomics for accurate identification of novel protease substrates.

**Table 1:** Selected legumain substrates and their cleavage sites identified in WT and legumain-deficient naïve mouse spleens by FAIMS-facilitated N-terminomics

Gene (UniProt)	Cleavage site: (P1 ↓ dimethylated peptide)	Subcellular localization	Log_2_(*Lgmn*^*−/−*^/WT)	−log_10_(p)
*Man2b1* (O09159)	N^424^ ↓ V^425^GPYGSGDSAPLQEAM AVLQHHDAVSGTAR	Lysosome	−3.2817	2.0668
*Tmpo* (Q61029)	N^58^ ↓ S^59^KGPPDFSSDEER	Nucleus	−6.1441	8.2222
*Tmpo* (Q61029)	N^58^ ↓ S^59^KGPPDFSSDEEREPTPVL GSGASVGR	Nucleus	−4.2837	3.0954
*Yars1* (Q91WQ3)	N^357^ ↓ S^358^EPEEVIPSR	Cytoplasm	−4.5893	5.3599

## Discussion

Protease substrate identification is crucial for understanding the complete proteolytic potential of proteases. Although conventional N-terminomics workflows provide sufficient data for *in vivo* substrate identification (19), they are hampered by difficult N-termini enrichment methods, which often compromise protein abundance information. Here, we developed and employed a novel N-terminomics workflow using FAIMS fractionation (40, 41) for the identification of both protein abundance and N-terminal alterations without the need for a dedicated N-terminal enrichment step. To overcome limited access to low abundance peptides, we utilized sample fractionation to broaden proteome coverage and allow improved detection of protease cleavage sites (neo-N-termini). While there are multiple alternative methods currently available (96, 97), we have employed FAIMS due to its hands-off, online application for reduced sample handling, streamlined preparation, and ability to reduce co-isolation of peptide precursors (98). We validated the effectiveness of FAIMS-facilitated N-terminomics analysis by benchmarking our approach against unfractionated cell lysates, affirming robust increases in peptide identification and total proteome coverage achieved by fractionating the samples (Figs. 1 and 2). Recently the HUNTER N-terminomics enrichment protocol was demonstrated to enable the isolation of >1000 N-termini from as little as 2 μg of starting material while >5000 N-termini could be identified using 200 μg coupled with pre-fractionation into 12 fractions prior to HUNTER enrichment (32). Though HUNTER-based enrichment may provide deeper N-termini coverage when >15x the starting material and offline fractionation is utilized, our approach identified >2500 N-termini can be identified using 12 μg of protein lysate from mouse spleen with the ability to simultaneously quantify protein abundance. These data, coupled with the reduced sample handling for LC-MS/MS analysis, validate FAIMS-facilitated N-terminomics as an effective strategy for deep proteome and N-terminome analyses, including identification of protein abundance changes and cleavage events.

Using FAIMS-facilitated N-terminomics, we investigated global proteome changes and physiological cleavage events in WT and *Lgmn*^*−/−*^ naïve spleen lysates, aiming to identify legumain substrates (Fig. 3, Fig. 4, Fig. 5). We chose to analyze murine spleens as legumain has been reported to be highly expressed in this tissue, yet its proteolytic impact has been largely unexplored (54, 77, 99). We identified a total of 6366 proteins, revealing global changes to several lysosomal cathepsins in the absence of legumain (Fig. 3). As with previous studies, this may suggest compensation for loss of lysosomal hydrolase activity in legumain-deficient samples, which may contribute to lysosomal storage disorders (56). Martínez-Fábregas and colleagues observed increased expression of lysosomal proteases and hydrolases in legumain-deficient kidneys (including cathepsin A, B, C, L, and X/Z), which is likely driven by STAT3 activation as a response to oxidative stress (57). In our study, we observed increased cathepsin B, L, E, and K expression in *Lgmn*^*−/−*^ spleens, which may signify tissue-specific responses. The 116 N-termini enriched in *Lgmn*^*−/−*^ spleens (Fig. 4*B*) likely reflect these altered proteolytic networks upon loss of legumain.

Considering cathepsins are involved in various immune processes such as toll-like receptor processing (100), inflammasome activation (101), and MHC-II invariant chain processing for antigen presentation (102), it is unsurprising we see an enrichment of proteins involved in immune-related pathways in *Lgmn*^*−/−*^ spleens (Fig. 3*H*). We also observed upregulation of several neutrophil-associated proteins, including neutrophil elastase and myeloperoxidase (Fig. 3, *B*–G). Previous studies have indicated increased populations of Gr-1^+^/Mac-1^+^ cells in *Lgmn*^*−/−*^ spleens, which may be the result of extramedullary hematopoiesis and splenomegaly (99). While we expect that the increased total abundance of neutrophil proteins is the result of increased neutrophil numbers, it remains to be investigated whether legumain also mediates cell-intrinsic effects within neutrophils.

To investigate legumain-dependent alterations of the physiological N-terminome, we used N-terminal dimethylation to chemically label N-terminal peptides, providing a chemical marker of proteolysis. Making use of the enhanced proteome depth afforded by FAIMS and recent advances in bioinformatics tools (*e.g.*, MSFragger), which utilizes an indexing-based approach to peptide searching that enables semi-tryptic searches with multiple variable modifications, we demonstrated that cleavage sites could be identified without direct enrichment of the N-termini. We observed 119 cleavage events enriched in WT tissue that correspond to cleavage after asparagine residues, representing potential legumain substrates (Supplemental Table S25). While other studies have interrogated the degradome of legumain (73, 74, 103), these studies were performed *in vitro* against a denatured proteome at low pH. Our study is the first to systematically profile the legumain substrate repertoire under native physiological conditions within tissue, potentially accounting for any differences in identified legumain substrates. While 93 of the cleavage sites contain P1 asparagine in both mouse and human proteins, it will be critical to consider the 26 divergent sites when translating legumain function in mouse models to human pathophysiology.

Intriguingly, only six of the identified cleavage events occurred within the endo-lysosomal system. This may reflect the rapid turnover of substrates in the lysosome, where legumain activity is optimal due to the low pH environment. This may also be the reason that we did not observe enrichment of cleavages after aspartic acid residues, which requires a low pH. The resulting fragments may also be short-lived due to secondary cleavages by other lysosomal proteases. The enrichment of asparagine in the P3 position of the non-P1 asparagine cleavages (Supplemental Fig. S17*E*) may hint at aminopeptidase activity following legumain cleavage. Cathepsin B has recently been characterized as a dipeptidyl carboxypeptidase with a preference to cleave substrates bearing asparagine in the P2′ position (104). We hypothesize that secondary cleavage by cathepsin B may obscure detection of legumain substrates in the lysosome. Inspired by recent work (37), we reanalyzed these data to reveal putative C-termini peptides in our FAIMS datasets (Supplemental Table S24, Supplemental Fig. S18). Asparagine was present in the P1 position of 45/110 WT-enriched C termini, reflecting putative legumain substrates. We also note the prevalence of asparagine enriched in the P2′ position of WT-enriched C-termini (Supplemental Fig. S19*C*), and this motif is absent in *Lgmn*^*−/−*^enriched C-termini (Supplemental Fig. S19*D*). While these results support the hypothesis that secondary processing by carboxypeptidases such as cathepsin B occurs following legumain cleavage, further C-terminomics analysis will be required to confirm this.

We identified 84 neo-N-termini (76%) with known localization to the nucleus or cytoplasm, suggesting a much broader substrate repertoire in these compartments than previously appreciated (Fig. 5*A*). From our data, we cannot confirm the subcellular location at which these proteins are cleaved; it is possible that the nuclear/cytoplasmic proteins are cleaved within lysosomes or elsewhere in the cell. Few studies have indicated a positive correlation between legumain and autophagy-related proteins such as ATG3 (105), and microtubule-associated protein 1A/1B-light chain 3 (LC3) (106). As such, increased legumain expression may associate with increased autophagic flux (107) and it may be that these observed “extra-lysosomal” cleavages are in fact mediated by autophagy. Nevertheless, the lack of identification of known lysosomal proteins, and the observation that cleavages arise from limited proteolysis and not degradation, provides support that the cleavages occur extra-lysosomally. Legumain localizes to the nucleus in the setting of colorectal cancer (52) and can cleave the nuclear protein FOXP3 in regulatory T-cells to inhibit T-cell differentiation (72). Cytoplasmic legumain is often associated with neurodegenerative phenotypes, where it cleaves tau (58), α-synuclein (108), and SET (109) to promote neurofibrillary tangles, plaque formation, and cognitive impairment. In the context of Alzheimer’s disease, legumain phosphorylation at S^226^ by SRPK2 led to accumulation of cytoplasmic legumain, promoting cleavage of tau, APP, and SRPK2 itself (110).

Although our data provide additional support that legumain can cleave substrates in neutral environments, how this occurs is still poorly understood. *In vitro*, cystatin E, an endogenous inhibitor of legumain, can bind and stabilize the active conformer at neutral pH by mimicking the C-terminal propeptide (111). Extracellular legumain can bind to α_v_β_3_ integrin through its RGD motif, which stabilizes its activity at neutral pH. Phosphorylation at S^226^ may also function to stabilize legumain in the cytoplasm or nucleus (110). In any case, we predict that legumain activity is lower in the nucleus or cytoplasm than in the lysosome. This slower cleavage may mediate limited proteolytic events, leading to longer lived products than in the degradative environment of the lysosome. We predict that many of these long-lasting cleavage products have concerted effects on protein function and cellular processes.

Amongst the putative legumain substrates that we identified, we validated cleavage of human cathepsin S at N^112^ ↓ R^113^ (N^120^ ↓ R^121^ in mouse), TMPO at N^58^ ↓ S^59^, and YARS1 at N^357^ ↓ S^358^ (Figs. 2, *G*–*I* and 6). As the majority of the proteins cleaved by legumain in mouse spleen (78%) contain an asparagine residue in the same position of the human homolog, we aimed to validate these cleavage events using human proteins to place them in the context of human physiology and disease. Cathepsin S is a lysosomal cysteine protease that is involved in antigen presentation (112) and contributes to diseases such as colitis (113), inflammation (114), and oral cancer (115). Various studies have previously reported legumain activity to be essential in the processing of several lysosomal cathepsins, including cathepsin B, L, D, and H (56, 57, 116). Our data suggest that legumain activity may also regulate the maturation of cathepsin S (Fig. 2). Interestingly, cleavage of mouse cathepsin S at N^120^ ↓ R^121^ was detected in RAW264.7 cells, but not spleen. In both datasets, cleavages at R^121^ ↓ T^122^ and T^122^ ↓ L^123^ were also detected. The latter is the predicted cleavage site for removal of the cathepsin S pro-peptide to facilitate its activation. These results suggest that in some contexts, legumain may directly mediate cathepsin S activation. As N^120^RT is a predicted site for *N*-linked glycosylation on mouse cathepsin S, we hypothesize that differential modification of this residue may dictate whether it can be cleaved by legumain. In human cathepsin S, however, the glycosylation site (N^104^IT) is distinct from the legumain cleavage site (N^112^ ↓ R^113^), suggesting differences in the interplay of these proteases across species. Further N-terminomics analysis of human samples may shed light on whether these observed cleavage events are similar between species.

TMPO is responsible for maintenance of the nuclear envelope and association with chromatin (117). We hypothesize that in the neutral environment of the nucleus, legumain may cleave TMPO in a controlled manner to initiate specific cellular effects. Caspase-3 and -6 have been implicated in processing TMPO (118, 119), resulting in release of chromatin from the nuclear envelope for degradation during early stages of apoptosis (120). Considering the identified N^58^ ↓ S^59^ cleavage is located between a LEM domain and the chromatin binding region, it is plausible that similar effects occur in the presence of legumain. The relationship between legumain and TMPO therefore requires further investigation.

YARS1 is a ligase that catalyzes the attachment of tyrosine to tRNA molecules (121). The legumain cleavage site on YARS1 (N^357^ ↓ S^358^) is located between its tRNA binding domain and nuclear localization signal (NLS) (Fig. 6, *G*–*I*). Considering both domains are required for Tyr-tRNA binding during protein synthesis (122), we hypothesize that legumain cleavage may lead to reduced tyrosine-tRNA ligase activity. The NLS may also become unmasked following legumain cleavage, thereby increasing the nuclear activities of YARS1 (121). Aminoacyl-tRNA synthetases are also known to be processed into fragments with cytokine potential. In the case of YARS1, matrix metalloproteinase (MMP)-mediated cleavage at S^386^ ↓ L^387^ and G^405^ ↓ L^406^ can enhance TLR2 signaling, TNF-α secretion from macrophages, and amplify monocyte/macrophage chemotaxis (123). YARS1 processing typically separates the N-terminal Rossman fold and C-terminal EMAPII domain (365–528 aa), yielding an N-terminal fragment (mini-TyrRS) known to promote endothelial cell migration and angiogenesis through transactivation of vascular endothelial growth factor receptor-2 (VEGFR2) (124, 125). N^357^ ↓ S^358^ was the only cleavage product identified in our spleen dataset, and it was 25-fold enriched in WT tissue compared to *Lgmn*^*−/−*^. Interestingly, this site was also enriched in inflamed skin sections from *Mmp2*^*−/−*^ mice when compared to WT (126). Considering legumain can cleave and activate pro-MMP-2 (70), legumain may be upregulated in MMP2-deficient mice to compensate its loss, and consequently, YARS1 is more processed. It is plausible that legumain cleavage at this site can mediate cytokine-like effects, but further study is required to test this.

In our *in vitro* cleavage assay, MAN2B1 exhibited a modest but detectible shift in mobility in response to legumain activity (Fig. 6*B*), supporting its cleavage proximal to the N- or C-terminus as opposed to the N^424^ cleavage event observed *in vivo* (Fig. 6, *A* and *B*). Intriguingly, human MAN2B1 is known to undergo post-translational processing into five smaller polypeptides (A-E). Cleavage of G^429^ ↓ S^430^ separates the A and B subunits (127). This site, along with N^424^ ↓ V^425^ and V^425^ ↓ G^426^ were significantly enriched in WT spleens and may suggest increased MAN2B1 processing in the presence of legumain with redundancy in the specific cleavage site (Supplemental Table S17). Considering processing into the ABC polypeptide occurs prior to separation of A and B subunits, it is plausible that legumain access may be blocked in our *in vitro* assay, where we instead observed cleavage at different sites (Fig. 6*B*). The presence of these processing events in the murine proteome, however, are yet to be elucidated and require further investigation.

As in previous studies, we observed differences in cleavage sites between *in vivo* and *in vitro* analyses (128). Differences in the cleavage sites identified between naïve spleen tissue and recombinant proteins may arise as a result of significant variances in the cleavage environment (129). In native conditions, legumain activity may be controlled by endogenous inhibitors, subcellular compartmentalization, and pH, limiting its interactions with substrates. *In vitro*, these conditions are unaccounted for, and as such, increased cleavage sites may be observed. Furthermore, as potential cofactors or conditions may be required for legumain substrate processing, we may also miss cleavage sites using *in vitro* assays, as these environments cannot be recapitulated. Additionally, considering neutral conditions result in the rapid destabilization of active recombinant legumain (51, 77), these *in vitro* assays are limited to acidic environments and therefore may not accurately capture more physiological cleavage events. While the *in vitro* cleavage assays are associated with a number of caveats and therefore should be carefully interpreted, we were nonetheless able to validate several of the cleavage sites identified *in vivo*.

In summary, we have validated the use of FAIMS-facilitated N-terminomics analysis for the robust and streamlined detection of protein abundance changes and cleavage events in WT and legumain-deficient mouse spleens. We identified a range of altered proteins including lysosomal cathepsins and neutrophil-associated proteins. Moreover, we provided the first comprehensive list of physiological legumain substrates identified using a systematic and unbiased approach, revealing novel insights into the proteolytic potential of legumain, especially outside of its lysosomal functions. These studies will assist in the delineation of the complete function of legumain in the cell and support efforts to develop legumain-targeted therapeutics for cancer and neurodegenerative diseases.

## Data Availability

The mass spectrometry proteomics data has been deposited in the Proteome Xchange Consortium *via* the PRIDE partner repository (130) with the data set identifiers PXD043136, PXD043124, PXD043122, PXD047734, and PXD047733.

## Supplemental data

This article contains supplemental data (91, 94).

## Conflict of interest

The authors declare that they have no conflicts of interest with the contents of this article.
