# Supplementary material for: Ion Mobility–Based Enrichment-Free N-Terminomics Analysis Reveals Novel Legumain Substrates in Murine Spleen
Source: Mol Cell Proteomics. 2024 Jan 8;23(2):100714. doi: 10.1016/j.mcpro.2024.100714 (PMC10862022; doi:10.1016/j.mcpro.2024.100714)
Supplement: Supplemental Data [file mmc1.pdf]

Ion mobility-based enrichment-free N-terminomics reveals novel legumain substrates in murine spleen

Alexander R. Ziegler[1], Antoine Dufour[2,3], Nichollas E. Scott[4#], & Laura E. Edgington-Mitchell[1#]

[1]Department of Biochemistry and Pharmacology, Bio21 Molecular Science and Biotechnology Institute, The University of Melbourne, Parkville, Victoria 3052, Australia.

[2]Department of Physiology and Pharmacology, University of Calgary, Calgary, Alberta T2N 1N4, Canada.

[3]McCaig Institute for Bone and Joint Health, University of Calgary, Calgary, Alberta T2N 1N4, Canada.

[4]Department of Microbiology and Immunology, Peter Doherty Institute, The University of Melbourne, Parkville, Victoria 3052, Australia.

Supplementary tables:

Table S1: Unfractionated protein data from SD134 and DMSO treated RAW264.7 cells without filtering for valid values in 3/4 biological replicates (MSFragger output)

Table S2: Unfractionated peptide data from SD134- and DMSO-treated RAW264.7 cells without filtering for valid values in 3/4 biological replicates (MSFragger output)

Table S3: Unfractionated protein data from SD134- and DMSO-treated RAW264.7 cells filtered for valid values in 3/4 biological replicates and with statistics (Perseus output)

Table S4: Unfractionated peptide data from SD134- and DMSO-treated RAW264.7 cells filtered for valid values in 3/4 biological replicates and with statistics (Perseus output)

Table S5: Unfractionated N-terminomics peptides (dimethylated N-terminus) from SD134- and DMSO-treated RAW264.7 cells filtered using R

Table S6: FAIMS-fractionated protein data from SD134- and DMSO-treated RAW264.7 cells without filtering for valid values in 3/4 biological replicates (MSFragger output)

Table S7: FAIMS-fractionated peptide data from SD134- and DMSO-treated RAW264.7 cells without filtering for valid values in 3/4 biological replicates (MSFragger output)

Table S8: FAIMS-fractionated protein data from SD134- and DMSO-treated RAW264.7 cells filtered for valid values in 3/4 biological replicates and with statistics (Perseus output)

Table S9: FAIMS-fractionated peptide data from SD134- and DMSO-treated RAW264.7 cells filtered for valid values in 3/4 biological replicates and with statistics (Perseus output)

Table S10: FAIMS-fractionated N-terminomics peptides (dimethylated N-terminus) from SD134- and DMSO-treated RAW264.7 cells filtered using R

Table S11: Putative legumain substrates identified in FAIMS-fractionated SD134- and DMSO-treated RAW264.7 cells

Table S12: Peptides from legumain-treated and untreated recombinant CTSS

Table S13: FAIMS-fractionated protein data from *Lgmn*$^{-/-}$ and wild-type naïve mouse spleens without filtering for valid values in 3/4 biological replicates (MSFragger output)

Table S14: FAIMS-fractionated peptide data from *Lgmn*$^{-/-}$ and wild-type naïve mouse spleens without filtering for valid values in 3/4 biological replicates (MSFragger output)

Table S15: FAIMS-fractionated protein data from *Lgmn*$^{-/-}$ and wild-type naïve mouse spleens filtered for valid values in 3/4 biological replicates and with statistics (Perseus output)

Table S16: FAIMS-fractionated peptide data from *Lgmn*$^{-/-}$ and wild-type naïve mouse spleens filtered for valid values in 3/4 biological replicates and with statistics (Perseus output)

Table S17: FAIMS-fractionated N-terminomics peptides (dimethylated N-terminus) from *Lgmn*$^{-/-}$ and wild-type naïve mouse spleens filtered using R

Table S18: STRING-db (v.11.5) analysis of 40 *Lgmn*$^{-/-}$ enriched proteins

Table S19: Enrichment analysis of FAIMS-fractionated peptides from *Lgmn*$^{-/-}$ and wild-type naïve mouse spleens (Perseus output)

Table S20: FAIMS-fractionated N-terminomics peptides significantly elevated in wild-type naïve mouse spleens and corresponding consensus motifs as analysed by TopFINDer

Table S21: FAIMS-fractionated N-terminomics peptides significantly elevated in *Lgmn*$^{-/-}$ naïve mouse spleens and corresponding consensus motifs as analysed by TopFINDer

Table S22: FAIMS-fractionated N-terminomics putative legumain substrates in naïve mouse spleens and corresponding consensus motifs as analysed by TopFINDer

Table S23: FAIMS-fractionated N-terminomics peptides significantly elevated in wild-type naïve mouse spleens with non-Asn cleavages and corresponding consensus motifs as analysed by TopFINDer

Table S24: Native and neo-C-termini identified in *Lgmn*$^{-/-}$ and wild-type naïve murine spleen

Table S25: Putative legumain substrates identified in FAIMS-fractionated *Lgmn*$^{-/-}$ and wild-type naïve mouse spleens

Table S26: STRING-db (v.11.5) analysis of 110 putative legumain substrate proteins

Table S27: Peptides from legumain-treated and untreated recombinant MAN2B1

Table S28: Peptides from legumain-treated and untreated recombinant TMPO

Table S29: Peptides from legumain-treated and untreated recombinant YARS1

Table S30: Unfractionated SD134 and DMSO treated RAW264.7 cells N-termini PSMs per experiment (MSFragger output)

Table S31: FAIMS-fractionated SD134- and DMSO-treated RAW264.7 cells N-termini PSMs per experiment (MSFragger output)

Table S32: FAIMS-fractionated *Lgmn*$^{-/-}$ and wild-type naïve mouse spleens N-termini PSMs per experiment (MSFragger output)

Table S33: Recombinant Proteins used for the *in vitro* cleavage assays

Table S34: Parallel reaction monitoring peptide inclusion list for *in vitro* cleavage assay N-terminomics analysis

Supplementary figures:

Fig. S1: SD-134 inhibits legumain activity in RAW264.7 cells

Fig. S2: Dimethylation efficacy of SD-134 (10 µM) and DMSO-treated RAW264.7 cell lysates

Fig. S3: Average percentage of N-termini from total peptides identified in each FAIMS fraction

Fig. S4: FAIMS-fractionation of RAW264.7 cell lysates improves proteome coverage of quantified N-termini

Fig. S5: FAIMS fractionation of RAW264.7 cell lysates enabled tighter distribution of LC-MS/MS data

Fig. S6: Comparison of N-termini identifications to a conventional N-terminomics workflow (TAILS)

Fig. S7: Comparison of Max label-free quantification (LFQ) values between unfractionated and FAIMS-fractionated RAW264.7 cell lysates

Fig. S8: Legumain directly processes cathepsin S *in vitro* as identified by FAIMS-enabled N-terminomics

Fig. S9: Legumain activity and expression are lost in legumain-deficient ($Lgmn^{-/-}$) naïve mouse spleen

Fig. S10: FAIMS-fractionated spleen lysate LC-MS/MS data demonstrates clustering at biological replicate level

Fig. S11: Dimethylation efficacy of wildtype (WT) and legumain-deficient ($Lgmn^{-/-}$) naïve mouse spleens

Fig. S12: Peptide properties of N-termini and non-N-termini identified in naïve spleen lysates from $Lgmn^{-/-}$ and WT mice

Fig. S13: FAIMS fractionation enables detection of unique peptides in each fraction

Fig. S14: Protein abundance changes observed in wild-type (WT) and legumain-deficient (*Lgmn*$^{-/-}$) spleen lysates are consistent across biological replicates

Fig. S15: Reactome pathway proteins are consistently upregulated in legumain-deficient (*Lgmn*$^{-/-}$) spleen lysates compared to wild-type (WT)

Fig. S16: N-termini resulting from asparaginyl cleavage exhibit significant missing quantifications in *Lgmn*$^{-/-}$ spleen lysates

Fig. S17: Proteolytic processing in murine spleens is mainly a result of endopeptidase activity

Fig. S18: Native and neo-C-termini identified in wild-type and legumain deficient (*Lgmn*$^{-/-}$) murine spleen confirm legumain cleavage events

Fig. S19: Native and neo-C-termini show similar cleavage motifs as identified N-termini in wild-type and legumain deficient (*Lgmn*$^{-/-}$) murine spleen

Fig. S20: Proteolytic products of identified putative legumain substrates are not differentially degraded following cleavage

Fig. S21: Legumain directly processes various proteins *in vitro* as identified by FAIMS-enabled N-terminomics

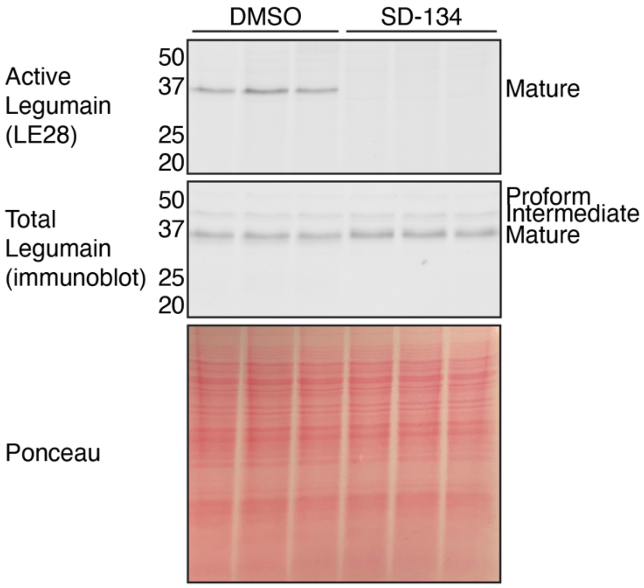

Fig. S1. SD-134 inhibits legumain activity in RAW264.7 cells. The legumain inhibitor SD-134 (10 μM) was added to RAW264.7 cells 16 hours prior to lysate labelling with the legumain-specific activity-based probe LE28 for 30 minutes (1 μM). In-gel fluorescence of legumain activity was detected by scanning for Cy5 fluorescence on a Typhoon 5 flatbed laser scanner (GE Healthcare). Total legumain was also detected by immunoblot. Ponceau S stain was used as a loading control.

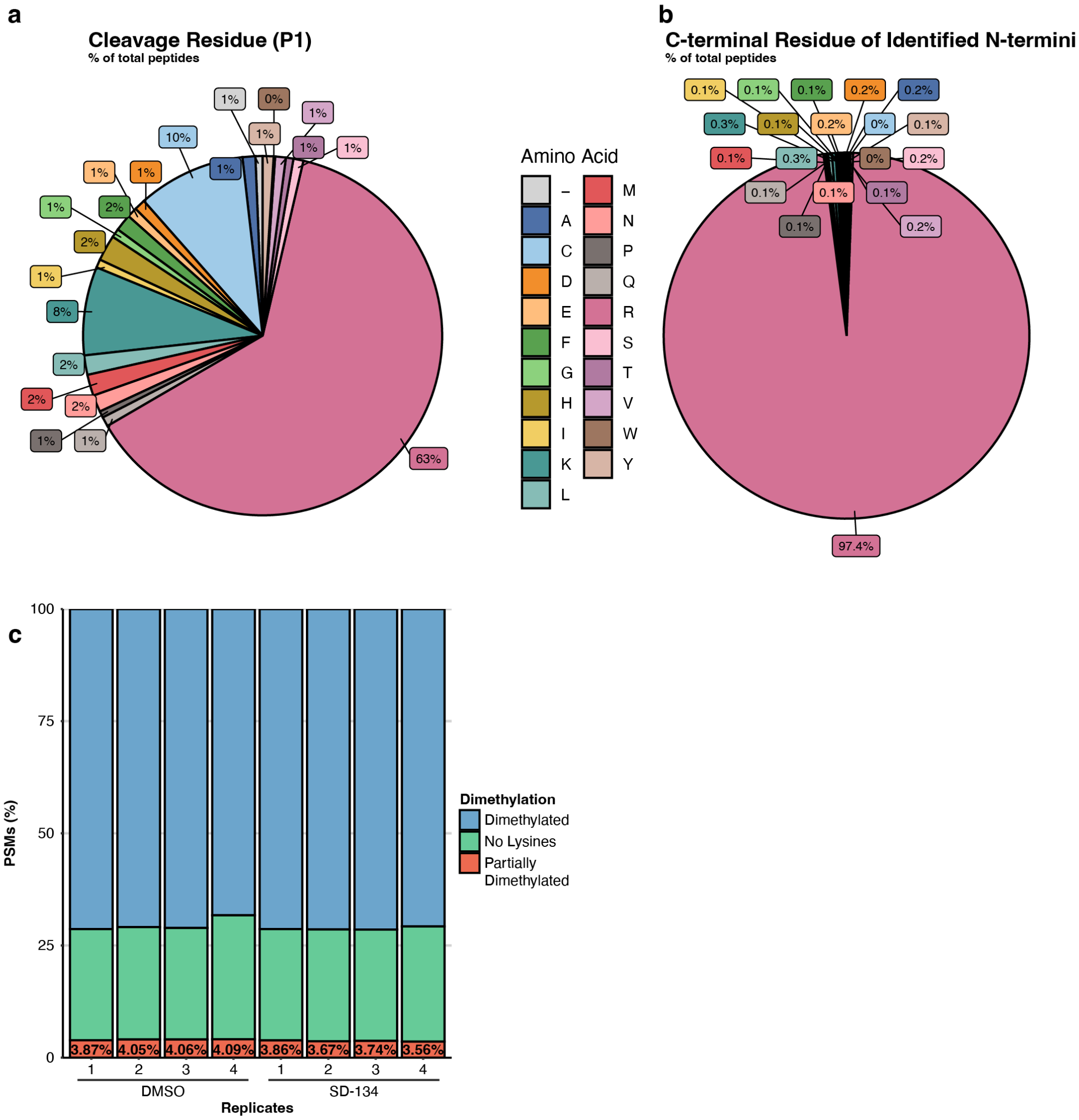

Fig. S2. Dimethylation efficacy of SD-134 (10 µM) and DMSO-treated RAW264.7 cell lysates. a-b. Cell lysates were denatured, reduced, and alkylated prior to N-terminal dimethylation by formaldehyde. Following LC-MS/MS analysis, amino acid residues prior to the identified peptide/P1 residue (**a**) and at the end of each identified peptide (**b**) were used as measures of dimethylation efficacy. **c.** Dimethylation status of each peptide was also analysed according to whether all lysines were dimethylated (blue), no lysines were present (green), or lysines were partially dimethylated (red) for each biological replicate.

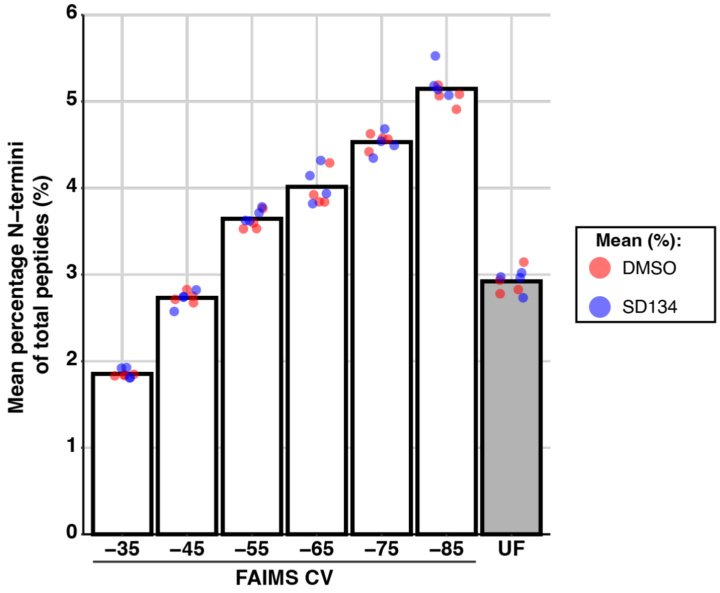

Fig. S3. Average percentage of N-termini from total peptides identified in each FAIMS fraction. Mean percentage of N-termini from total peptides identified were plotted per FAIMS compensational voltage (CV). Each data point represents a biological replicate (DMSO = red, SD134 = blue). Mean percentage for unfractionated (UF) RAW264.7 cell lysates are also shown (grey).

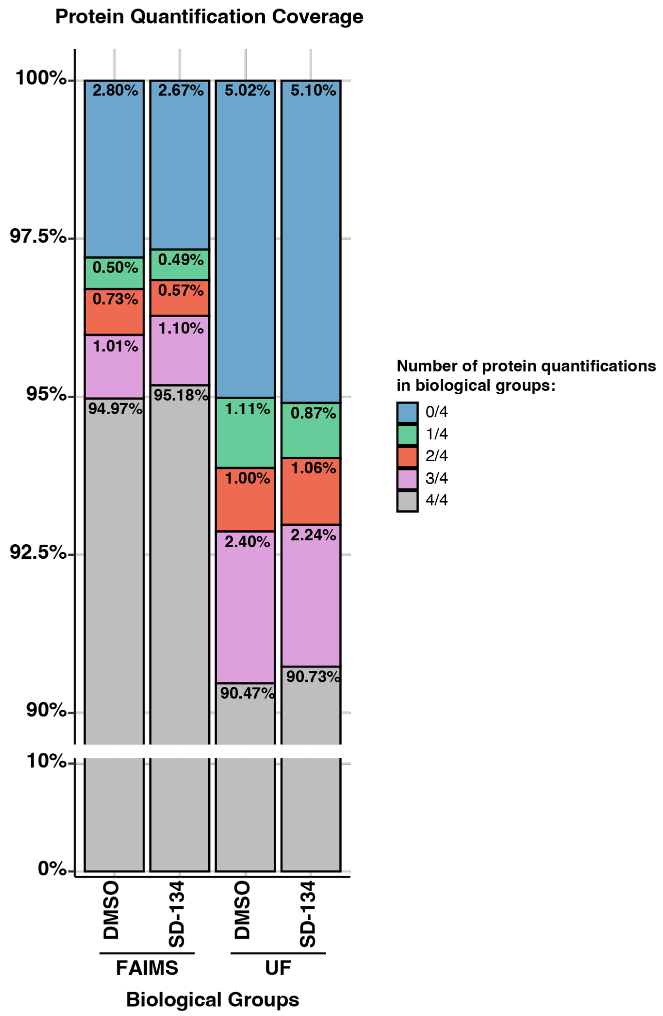

Fig. S4. FAIMS-fractionation of RAW264.7 cell lysates improves proteome coverage of quantified N-termini. Following LC-MS/MS analysis, data were matched against a murine database in MSFragger for identification and quantification of proteins and N-termini. Quantified N-termini were assessed per biological group in R (v.4.2.0) as to whether they contained protein quantifications in each biological replicate. Zoom shows range from 90-100%.

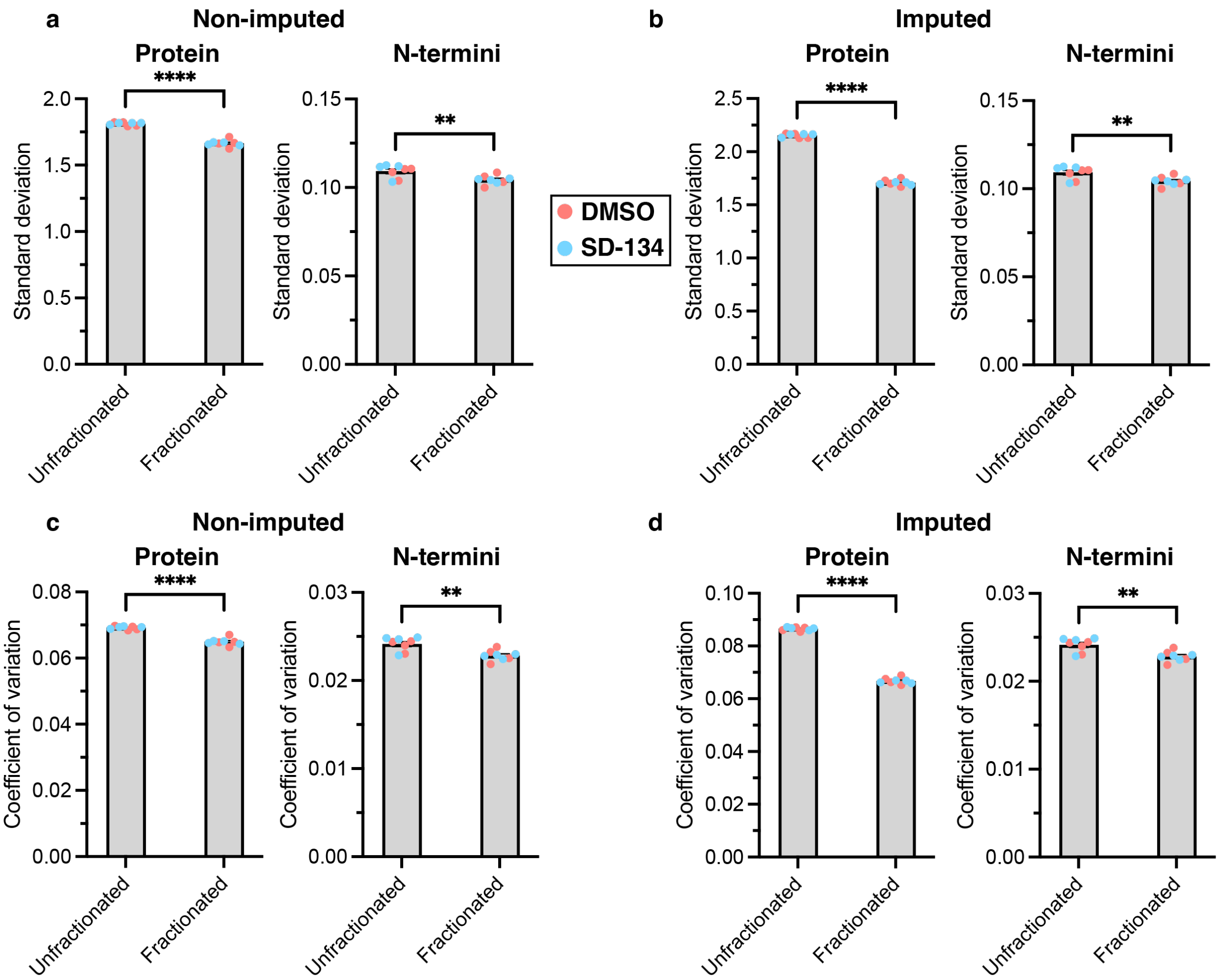

Fig. S5. FAIMS fractionation of RAW264.7 cell lysates enabled tighter distribution of LC-MS/MS data. a-d. Following LC-MS/MS analysis of FAIMS-fractionated SD-134 (blue) and DMSO (red) treated RAW264.7 cell lysates, data files were processed in Perseus (v.1.6.0.7) to include at least three of four valid values in at least one of the groups (n = 4/group). Standard deviations (**a-b**) and coefficient of variations (**c-d**) of $\log_2$(DMSO/SD-134) were calculated for each biological replicate on the protein and N-termini level for both non-imputed (**a, c**) and imputed (**b, d**) data in Perseus (v.1.6.0.7). Imputed values were based on a normal distribution with σ-width = 0.3 and σ-downshift = -1.8. A student's t-test was used for pairwise comparisons (*p<0.05, **p<0.01, ***p<0.001, ****p<0.0001).

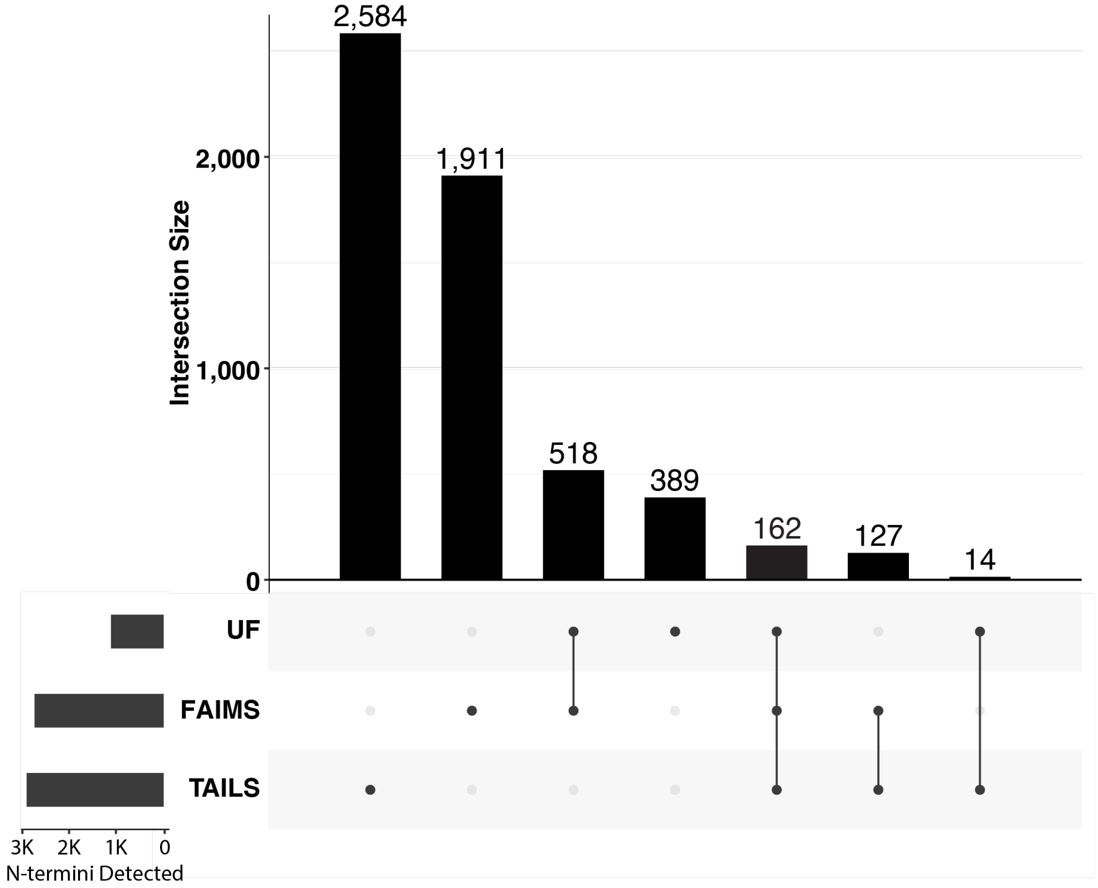

Fig. S6. Comparison of N-termini identifications to a conventional N-terminomics workflow (TAILS). Overlap of N-termini observed between unfractionated, FAIMS-fractionated, and TAILS (Terminal Amine Isotopic Labelling of Substrates, data obtained from Anderson et al. (2020)) methods. Bottom-left panel shows total N-termini detected in each experiment. UF=unfractionated. The TAILS experiment was performed as followed; RAW264.7 cells were treated with DMSO or 100 µM legumain-specific inhibitor LI-1 (n =4). Following labelling with light and heavy formaldehyde, N-termini were negatively selected for using a dendritic polyglycerol aldehyde polymer for LC-MS/MS analysis on the Orbitrap Fusion Lumos Tribrid mass spectrometer. LC-MS/MS files were searched against the murine proteome database using MaxQuant (v.1.6.0.1) for peptide-spectrum matching.

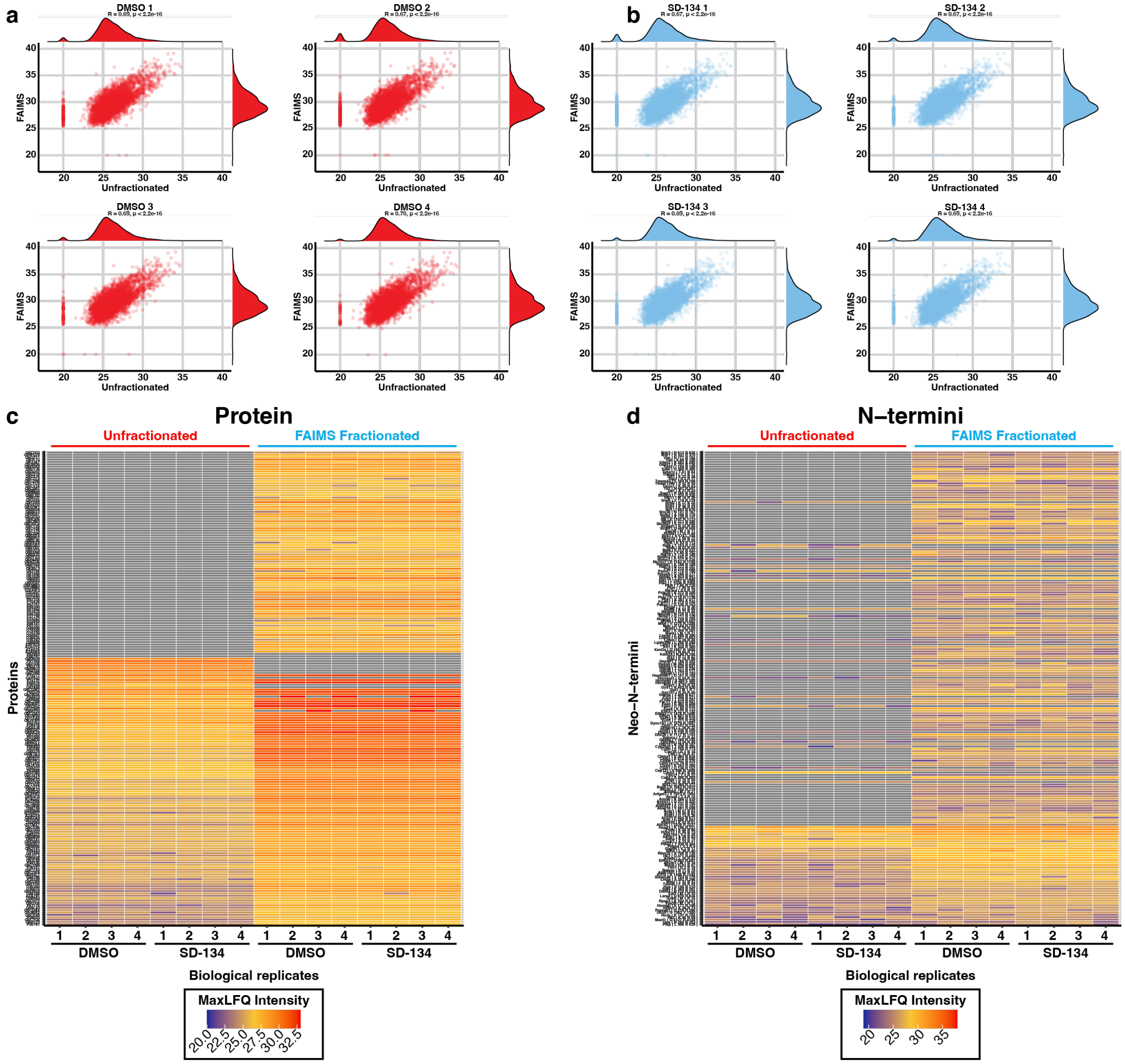

Fig. S7. Comparison of Max label-free quantification (LFQ) values between unfractionated and FAIMS-fractionated RAW264.7 cell lysates. a-b. Identified proteins with valid non-imputed MaxLFQ intensity values in both unfractionated (x-axis) and FAIMS-fractionated (y-axis) data were plotted per biological replicate (n = 4/group, DMSO and SD-134). Invalid values were assigned an arbitrary value of 20 for visualisation on the plot. Pearson correlation coefficients were calculated per biological replicate using the ggpubr package in R (v.4.2.0). **c-d**. MaxLFQ intensity values at the protein (**c**) and dimethylated N-termini (**d**) level were shown as heatmaps. Random sampling of 200 protein or N-termini was used. Missing values are shown in grey.

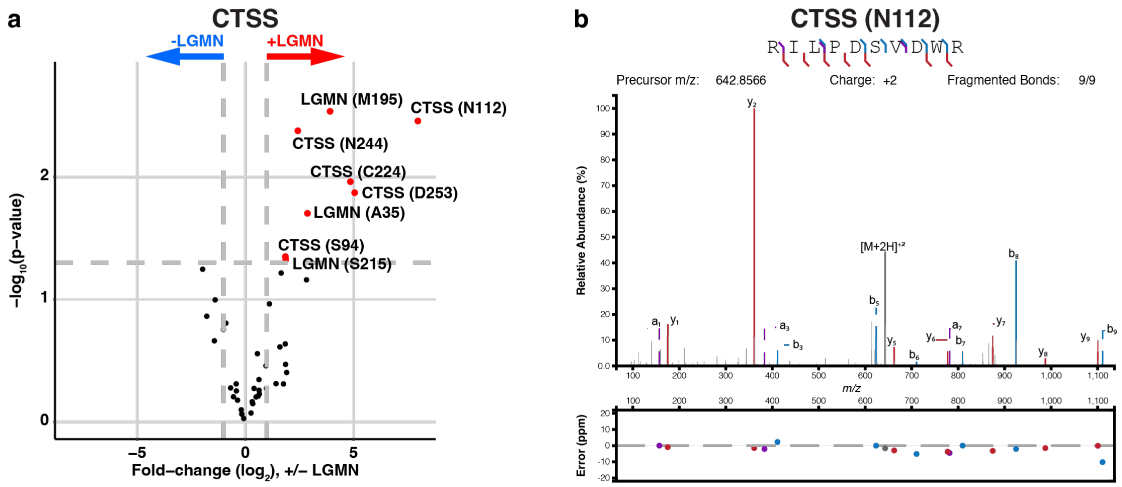

Fig. S8. Legumain directly processes cathepsin S *in vitro* as identified by FAIMS-enabled N-terminomics. a-b. Recombinant proteins were incubated with activated recombinant legumain (LGMN) prior to N-terminomics analysis (n=4/group). **a.** Data were filtered to contain valid quantifications in ≥ 3 of 4 replicates in at least one of the groups (+/- LGMN) and statistics were performed using Perseus (v.1.6.0.7) using a two-sample t-test. All dimethylated N-termini were visualised by volcano plot **b.** MS2 analysis confirmed the dimethylation of the CTSS peptide ^{113}RILPDSVDWR^{122} within legumain-treated samples supporting its cleavage. Figures were created using http://www.interactivepeptidespectralannotator.com/PeptideAnnotator.html with a fragment tolerance of ±10 ppm.

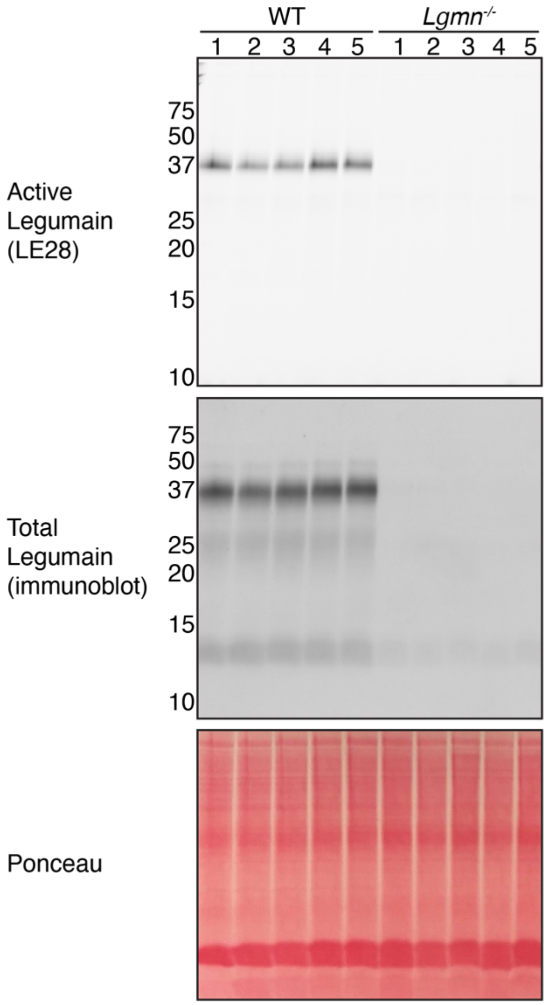

Fig. S9. Legumain activity and expression are lost in legumain-deficient (*Lgmn*$^{-/-}$) naïve mouse spleens. Spleens were lysed and labelled with the legumain-specific activity-based probe LE28 (1 µM) prior to SDS-PAGE analysis. In-gel fluorescence was imaged with the Cy5 filter of the Typhoon 5 flatbed laser scanner (GE Healthcare). Total legumain was also detected by immunoblot. Ponceau S stain was used as a loading control.

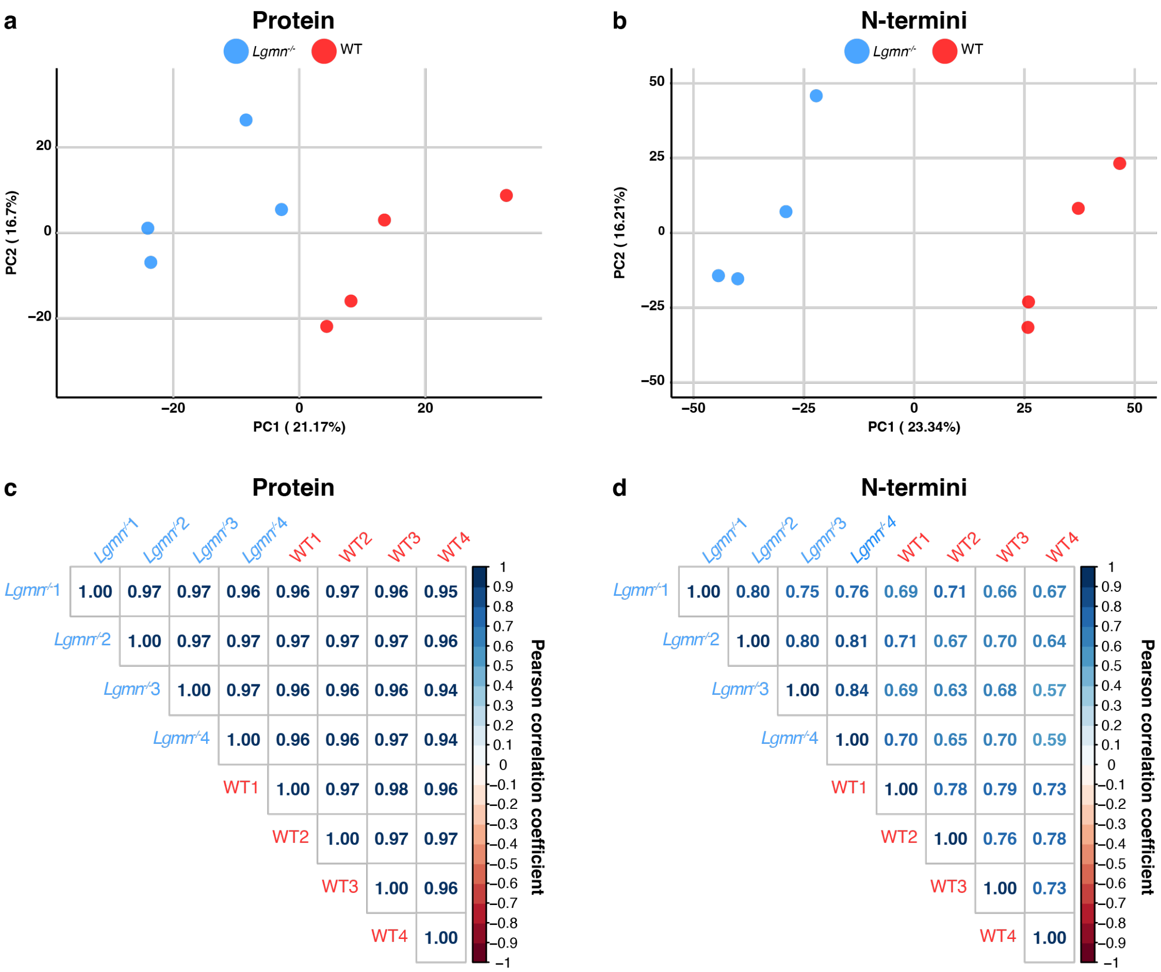

	Lgmn-/-1	Lgmn-/-2	Lgmn-/-3	Lgmn-/-4	WT1	WT2	WT3	WT4
Lgmn-/-1	1.00	0.97	0.97	0.96	0.96	0.97	0.96	0.95
Lgmn-/-2		1.00	0.97	0.97	0.97	0.97	0.97	0.96
Lgmn-/-3			1.00	0.97	0.96	0.96	0.96	0.94
Lgmn-/-4				1.00	0.96	0.96	0.97	0.94
WT1					1.00	0.97	0.98	0.96
WT2						1.00	0.97	0.97
WT3							1.00	0.96
WT4								1.00

	Lgmn-/-1	Lgmn-/-2	Lgmn-/-3	Lgmn-/-4	WT1	WT2	WT3	WT4
Lgmn-/-1	1.00	0.80	0.75	0.76	0.69	0.71	0.66	0.67
Lgmn-/-2		1.00	0.80	0.81	0.71	0.67	0.70	0.64
Lgmn-/-3			1.00	0.84	0.69	0.63	0.68	0.57
Lgmn-/-4				1.00	0.70	0.65	0.70	0.59
WT1					1.00	0.78	0.79	0.73
WT2						1.00	0.76	0.78
WT3							1.00	0.73
WT4								1.00

Fig. S10. FAIMS-fractionated spleen lysate LC-MS/MS data demonstrates clustering at biological replicate level. a-b. Principal component analysis (PCA) of proteins (**a**) and N-termini (**b**) identified using FAIMS-enabled N-terminomics was performed. Wild-stype (WT) data are shown in red and legumain-deficient (*Lgmn*$^{-/-}$) data in blue (n = 4/group). **c-d.** Max label-free quantification values per biological replicate were analysed for Pearson correlation coefficient in Perseus (v.1.6.0.7) and visualised as a correlogram using the corrplot package in the R statistical environment.

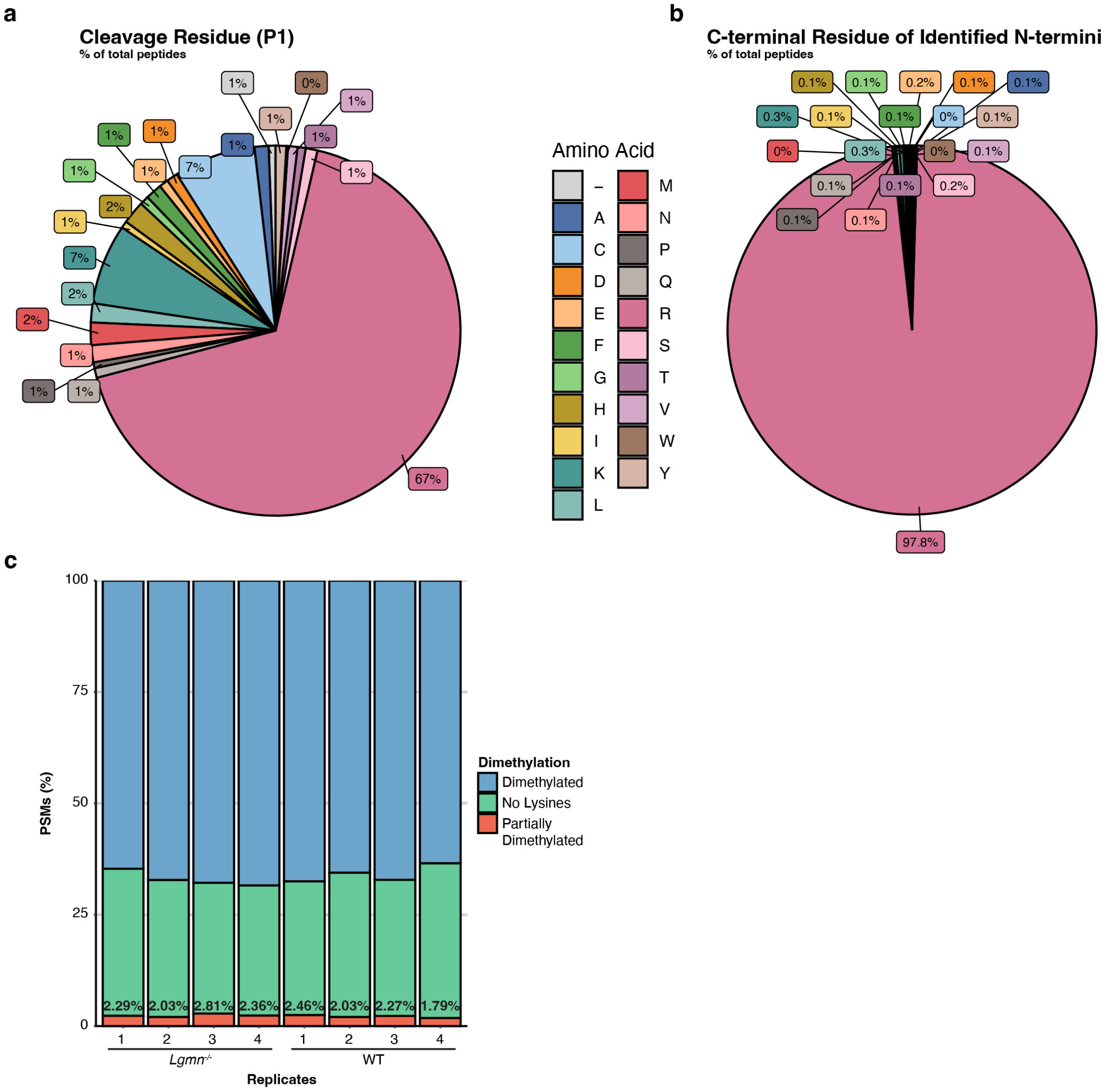

Fig. S11. Dimethylation efficacy of wildtype (WT) and legumain-deficient (*Lgmn*$^{-/-}$) naïve mouse spleens. a-b. Spleen lysates were denatured, reduced, and alkylated prior to N-terminal dimethylation by formaldehyde. Following LC-MS/MS analysis, amino acid residues prior to the identified peptide/P1 residue (**a**) and at the end of each identified peptide (**b**) were used as measures of dimethylation efficacy. **c.** Dimethylation status of each peptide was also analysed according to whether all lysines were dimethylated (blue), no lysines were present (green), or lysines were partially dimethylated (red) for each biological replicate.

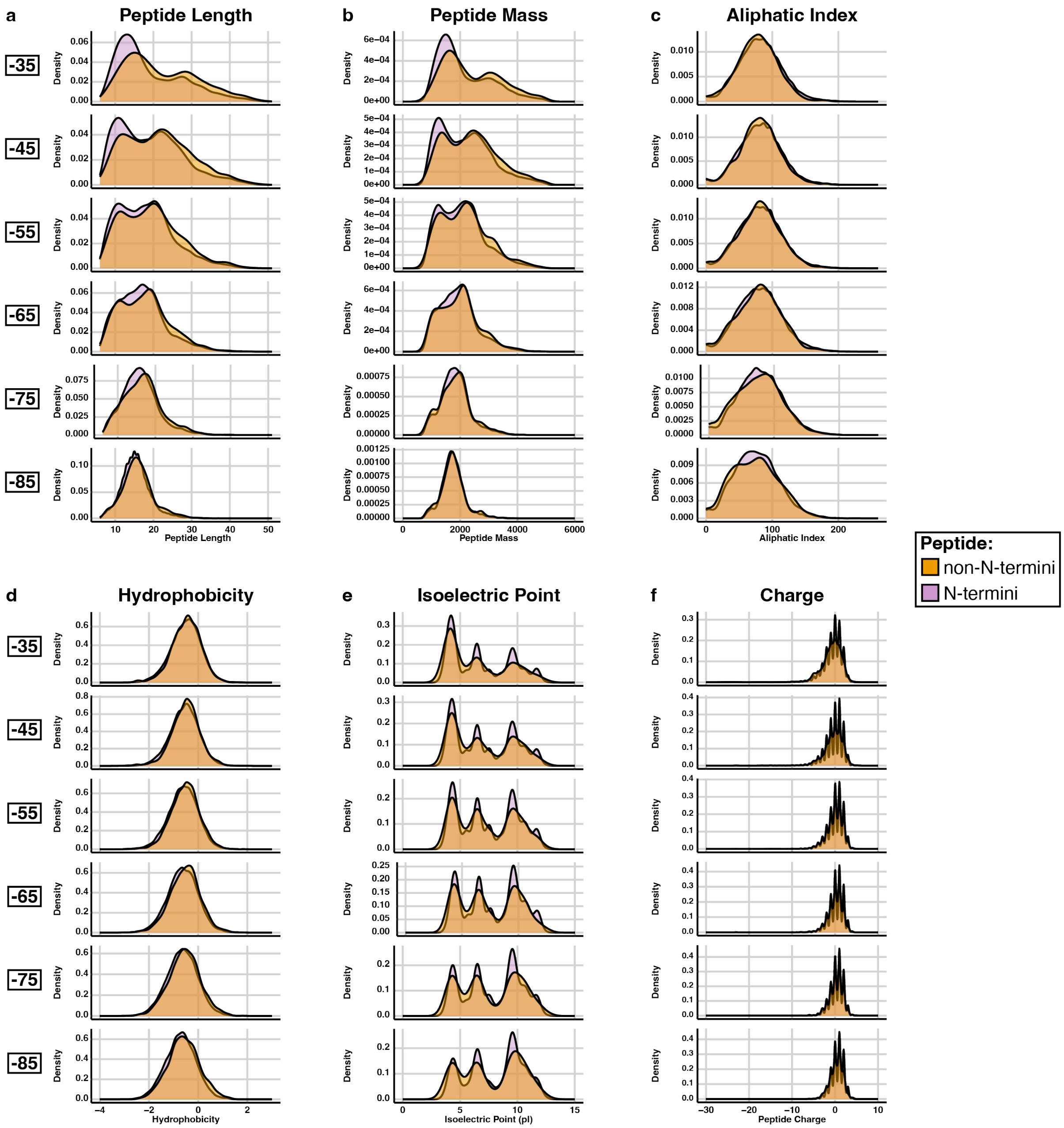

Fig. S12. Peptide properties of N-termini and non-N-termini identified in naïve spleen lysates from *Lgmn*$^{-/-}$ and WT mice. a-f. Tryptic peptides (orange) and dimethylated peptides (purple)-spectrum matches in each FAIMS fraction were analysed according to the indicated peptide properties and plotted using the Peptide package (v.2.4.5) with default settings in R (v.4.2.0).

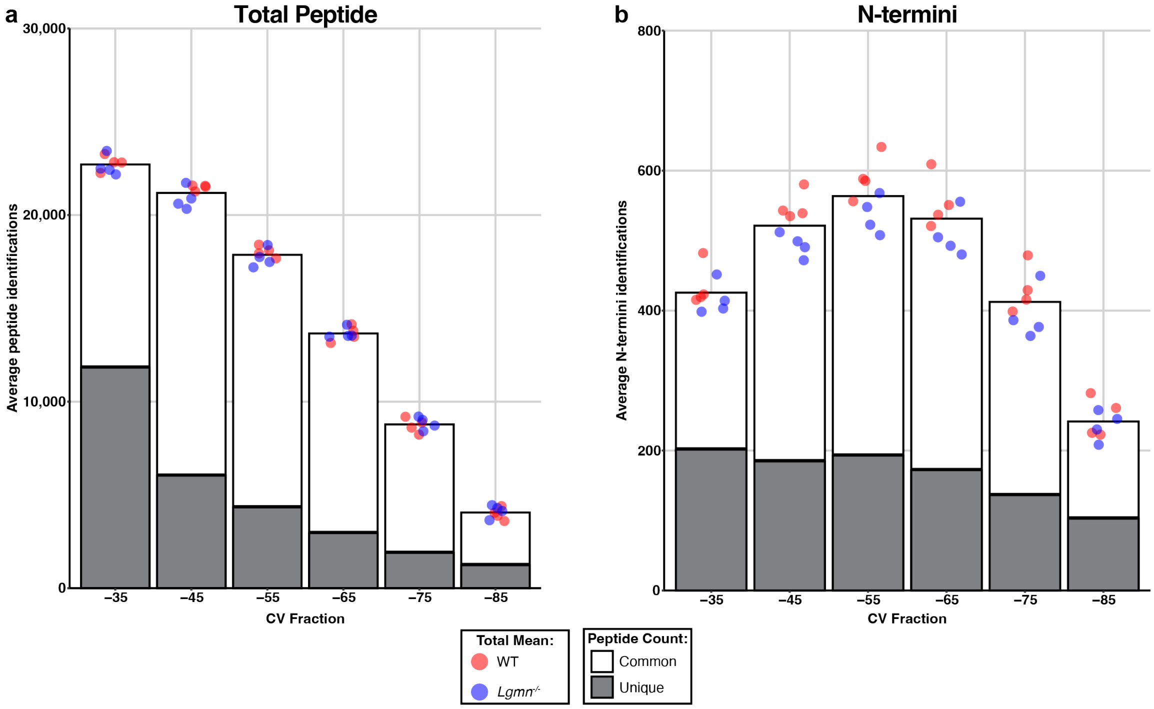

Fig. S13. FAIMS fractionation enables detection of unique peptides in each fraction. a-b. Spleen lysates were prepared for mass spectrometry analysis on an Orbitrap 480™ mass spectrometer coupled to a FAIMS (high-field asymmetric waveform ion mobility spectrometry) device. Each biological replicate was fractionated into six individual fractions based on compensational voltage (CV) of -35, -45, -55, -65, -75, or -85. Following peptide-database matching using MSFragger (Fragpipe v.18.0), average number of identifications per biological replicate was visualised at the total peptide (**a**) and N-termini (**b**) level (n =4/group). Peptides and N-termini identified in only one CV fraction are shown in grey (unique), whilst those identified in more than one CV fraction are shown in white (common). Data points represent biological replicates where red indicates wildtype (WT and blue indicates legumain-deficient (*Lgmn*$^{-/-}$) spleen lysates.

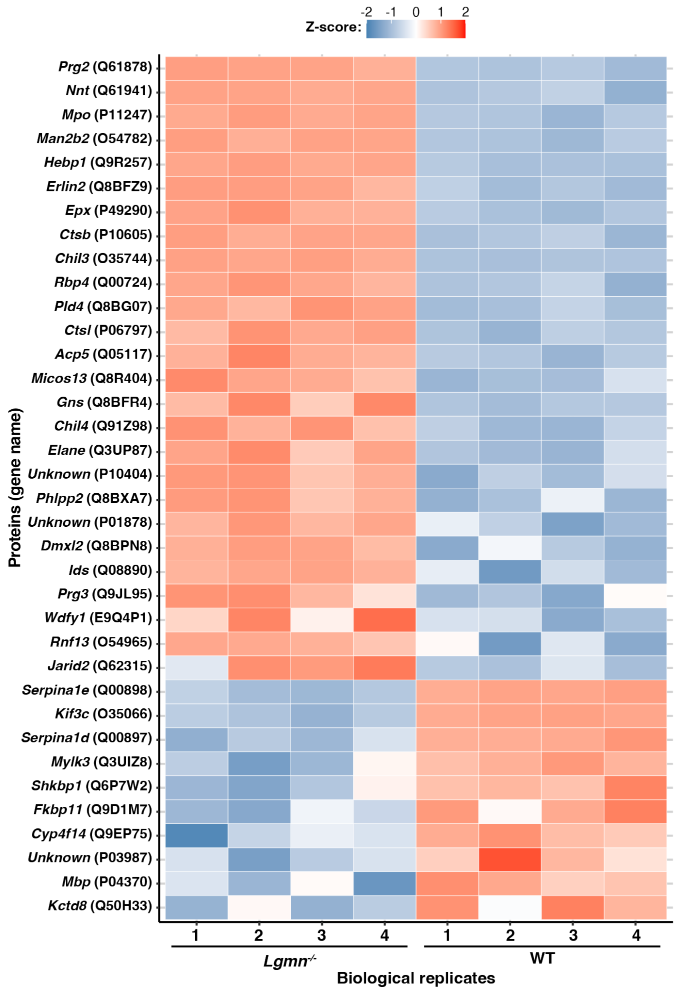

Fig. S14. Protein abundance changes observed in wild-type (WT) and legumain-deficient (*Lgmn*$^{-/-}$) spleen lysates are consistent across biological replicates. Significantly enriched proteins (abs ($\log_2$(WT/*Lgmn*$^{-/-}$)) > 1 and -log10(p) > 2) were visualised on a heatmap. Z-scores were calculated based on the max label-free quantification (LFQ) intensity for each protein. Red indicates increased intensity from the mean and blue indicates decreased intensity from the mean. See Supplementary Table 15 for the complete list.

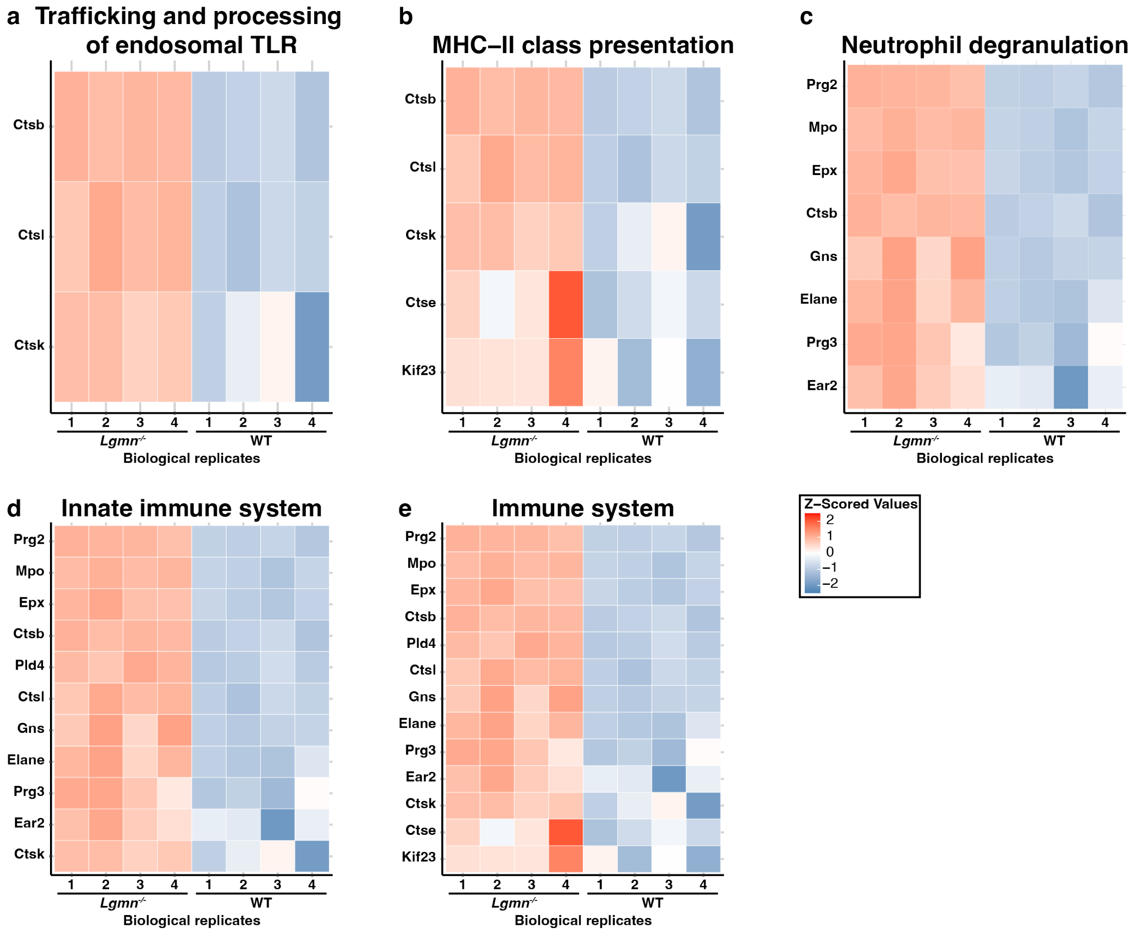

Fig. S15. Reactome pathway proteins are consistently upregulated in legumain-deficient (*Lgmn*$^{-/-}$) spleen lysates compared to wild-type (WT). a-e. Following LC-MS/MS analysis of spleen lysates (n = 4/group), data were searched against an unreviewed mouse database in MSFragger (Fragpipe v.18.0) and quantified. A student's two-way t-test was performed in Perseus (v.1.6.0.7) to determine significant protein abundance changes between *Lgmn*$^{-/-}$ and WT spleen lysates. Proteins significantly upregulated in *Lgmn*$^{-/-}$ spleens ($\log_2$(WT/*Lgmn*$^{-/-}$)>1 and $-\log_{10}$(p)>1.3) were further analysed by STRING-dp for reactome pathways. Proteins included in each identified reactome pathway were extracted and max label-free quantification values for each biological replicate were used to calculate the z-score and deviation of each replicate from the mean (red = increased, blue = decreased). These values for each of the proteins are visualised as heatmaps.

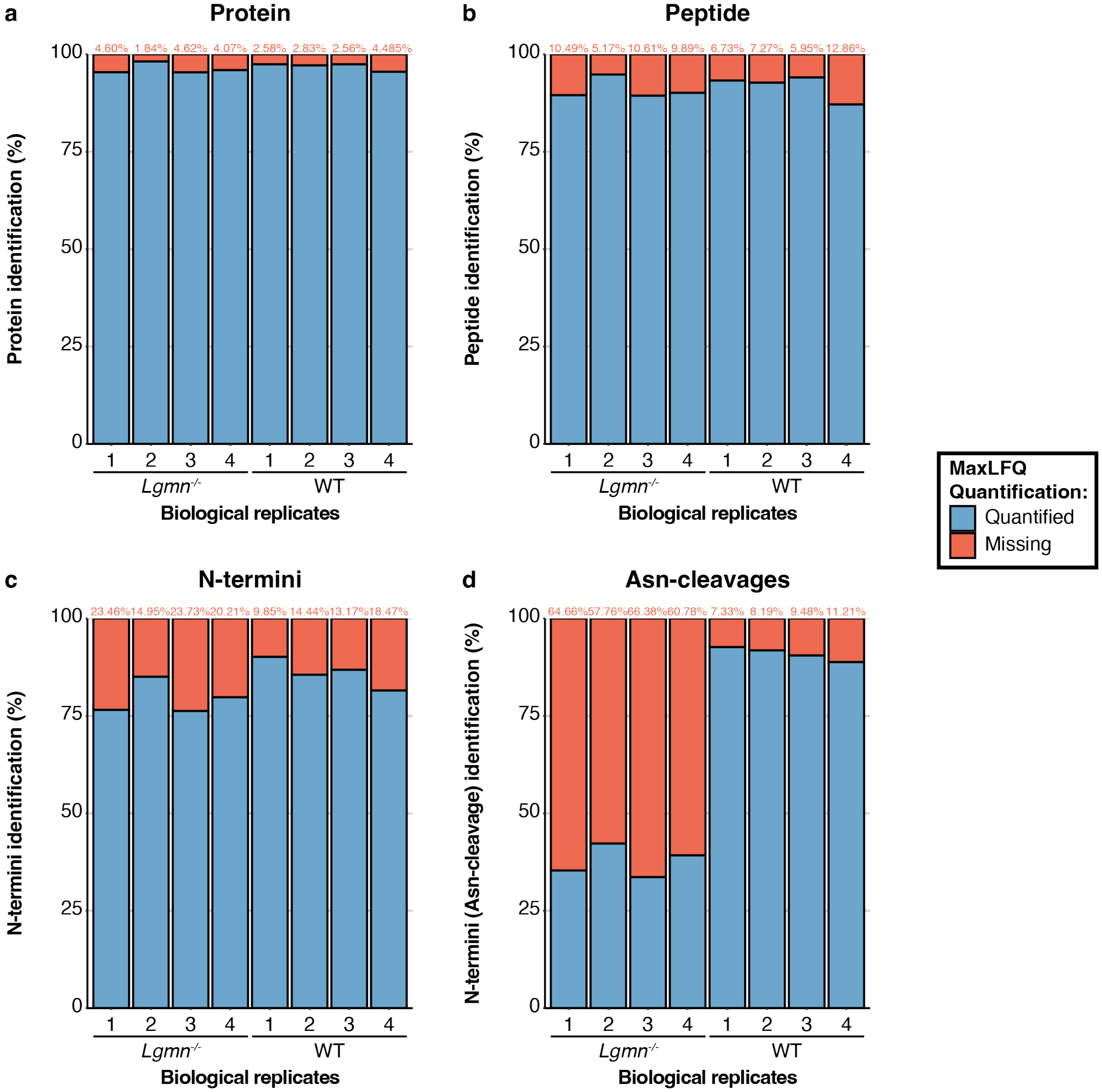

Fig. S16. N-termini resulting from asparaginyl cleavage exhibit significant missing quantifications in *Lgmn*$^{-/-}$ spleen lysates. a-d. Following protein (**a**) and peptide (**b-d**) quantification, data were assessed for percentage of missing quantifications per biological replicate. Peptide quantifications were further filtered for dimethylated N-termini representing native and protease-generated neo-N-termini (**c**). N-termini arising from asparaginyl (Asn) cleavage were identified for as potential neo-N-termini generated by legumain processing (**d**). Identifications with MaxLFQ quantification in biological replicates are shown in blue whilst missing quantifications are in red.

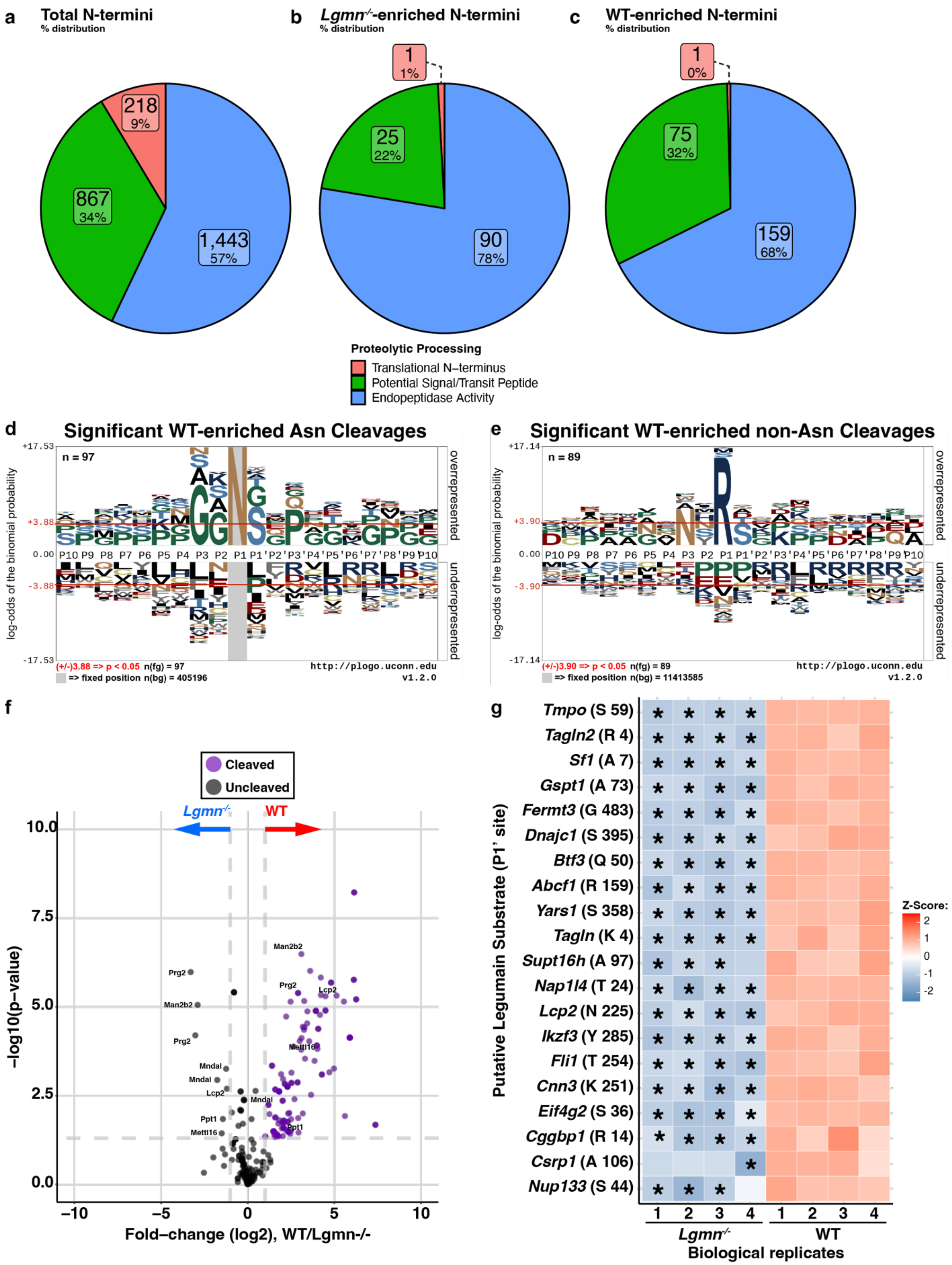
a
Total N-termini
% distribution
218
9%
867
34%
1,443
57%
b
Lgmn-/--enriched N-termini
% distribution
1
1%
25
22%
90
78%
c
WT-enriched N-termini
% distribution
1
0%
75
32%
159
68%
Proteolytic Processing
Translational N–terminus
Potential Signal/Transit Peptide
Endopeptidase Activity
d
Significant WT-enriched Asn Cleavages
n = 97
log-odds of the binomial probability
overrepresented
underrepresented
(+/-)3.88 => p < 0.05 n(fg) = 97
=> fixed position n(bg) = 405196
http://plogo.uconn.edu
v1.2.0
e
Significant WT-enriched non-Asn Cleavages
n = 89
(+/-)3.90 => p < 0.05 n(fg) = 89
=> fixed position n(bg) = 11413585
http://plogo.uconn.edu
v1.2.0
f
Cleaved
Uncleaved
Lgmn-/-
WT
−log10(p−value)
Fold−change (log2), WT/Lgmn-/-
Man2b2
Prg2
Lcp2
Mettl16
Mndal
Ppt1
g
Putative Legumain Substrate (P1' site)
Tmpo (S 59)
Tagln2 (R 4)
Sf1 (A 7)
Gspt1 (A 73)
Fermt3 (G 483)
Dnajc1 (S 395)
Btf3 (Q 50)
Abcf1 (R 159)
Yars1 (S 358)
Tagln (K 4)
Supt16h (A 97)
Nap1l4 (T 24)
Lcp2 (N 225)
Ikzf3 (Y 285)
Fli1 (T 254)
Cnn3 (K 251)
Eif4g2 (S 36)
Cggbp1 (R 14)
Csrp1 (A 106)
Nup133 (S 44)
Z-Score:
Lgmn-/-
WT
Biological replicates

Fig. S17. Proteolytic processing in murine spleens is mainly a result of endopeptidase activity. a-c. Following LC-MS/MS analysis of FAIMS-fractionated spleen lysates (n = 4/group), each identified N-termini was separated based on the position of the identified cleavage site in the protein where red indicates the translation N-terminus (1-2 aa), green indicates potential signal or transit peptides (3-65 aa) and blue indicates endopeptidase activity (66+ aa). **d-e.** Sequence motifs of N-termini significantly enriched in WT spleen lysates with asparaginyl (n = 97, putative legumain substrates) (**d**) or non-asparaginyl (n = 90) (**e**) cleavages were created using plogo (O'Shea et al. 2013). Overrepresented amino acids appear above and underrepresented below the x-axis ($p < 0.05$). **f.** Neo-N-termini arising from asparaginyl cleavage and significantly enriched in WT spleen lysates ($\log_2(Lgmn^{-/-}/\text{WT}) < -1$ and $-\log_{10}(p) > 1.3$), and their uncleaved counterpart were visualised as a volcano plot. Of the 235 WT-enriched neo-N-termini, 128 had corresponding uncleaved fully tryptic peptides identified. The neo-N-termini (cleaved) are shown in purple, and the fully tryptic (uncleaved) peptides are shown in black. **g.** Top 20 legumain substrates identified are represented as a heat map. Z-scores were calculated based on the max label-free quantification (LFQ) intensities for each N-terminus. Red indicates increased intensity from the mean and blue indicates decreased intensity from the mean. Gene names and the identified N-termini are shown. Values which were imputed from a normal distribution (σ-width = 0.3 and σ-downshift = -1.8) are indicated by an asterisk (*).

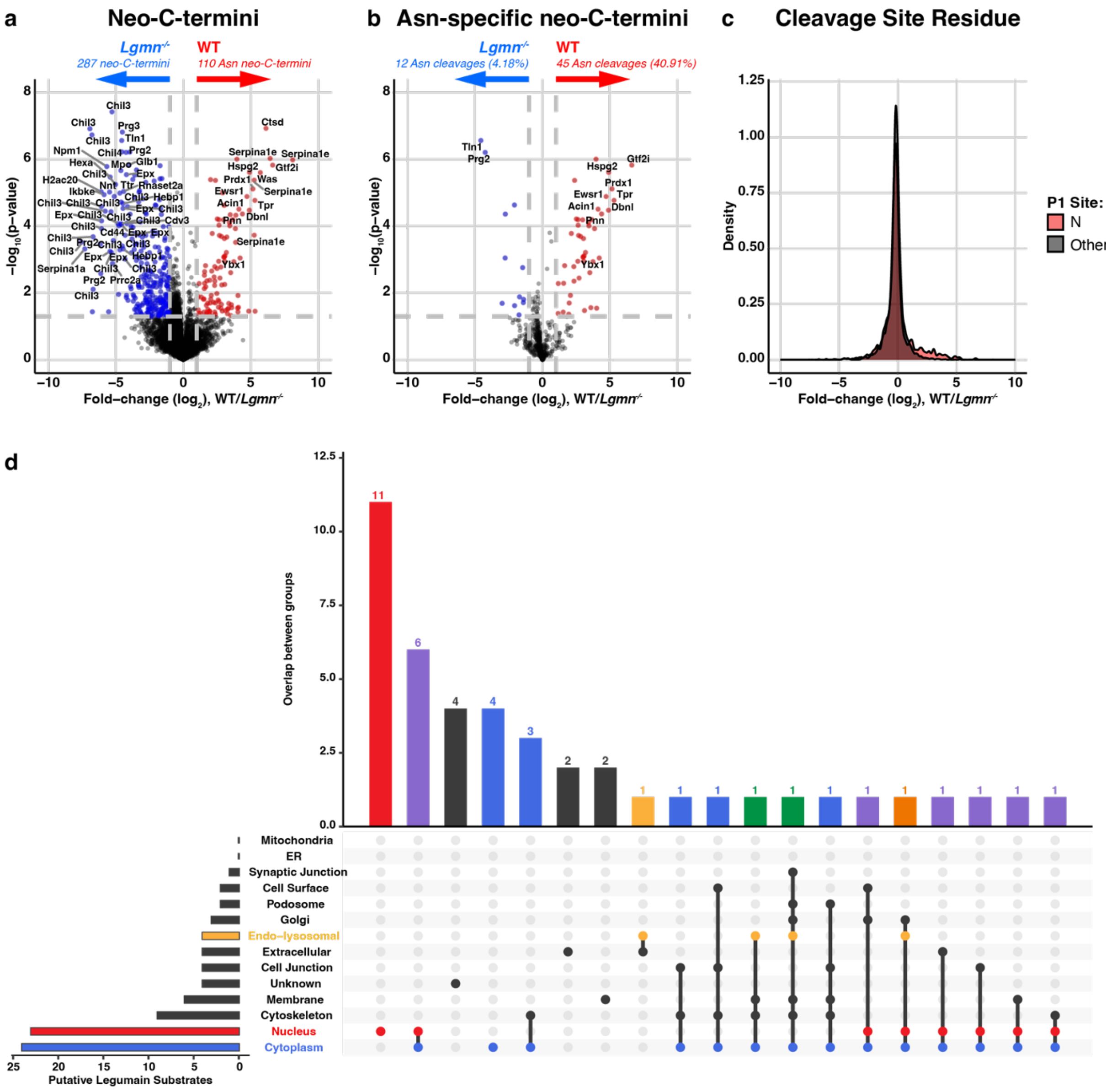

Fig. S18. Native and neo-C-termini identified in wild-type and legumain deficient (*Lgmn$^{-/-}$*) murine spleen confirm legumain cleavage events. a-c. Identified peptides were filtered for those present in at least 3 of 4 biological replicates in at least one group (n = 4/group). Peptides were bioinformatically filtered for C-termini by keeping ArgC specificity at the N-terminus and non-ArgC specificity at the C-terminus of the peptide sequence. C-termini were further filtered as either "native" or "neo". A two-sample t-test was performed and neo-C-termini were visualised by volcano plot. **a.** WT-enriched neo-C-termini are shown in red ($\log_2$(WT/*Lgmn$^{-/-}$*) > 1 and -$\log_{10}$(p) > 1.3) and *Lgmn$^{-/-}$* in blue ($\log_2$(WT/*Lgmn$^{-/-}$*) < -1 and -$\log_{10}$(p) > 1.3). **c.** Asparaginyl cleavage events were also identified. **d.** Density plot based on cleavage site residue of data shown in **Fig. S18a** showing $\log_2$(WT/*Lgmn$^{-/-}$*) distribution of neo-C-termini. **d.** Upset plot of subcellular localisations of the 45 WT-enriched asparaginyl neo-C-termini identified. Information is taken from UniProt (https://www.uniprot.org/). Compartments of interest are highlighted such that red indicates

nucleus, blue indicates cytoplasm, and yellow indicates endo-lysosomal system. Proteins localised to both nuclear and cytoplasmic regions are highlighted in purple, both cytoplasmic and endo-lysosomal in green, and all three nuclear, cytoplasmic, endo-lysosomal in orange.

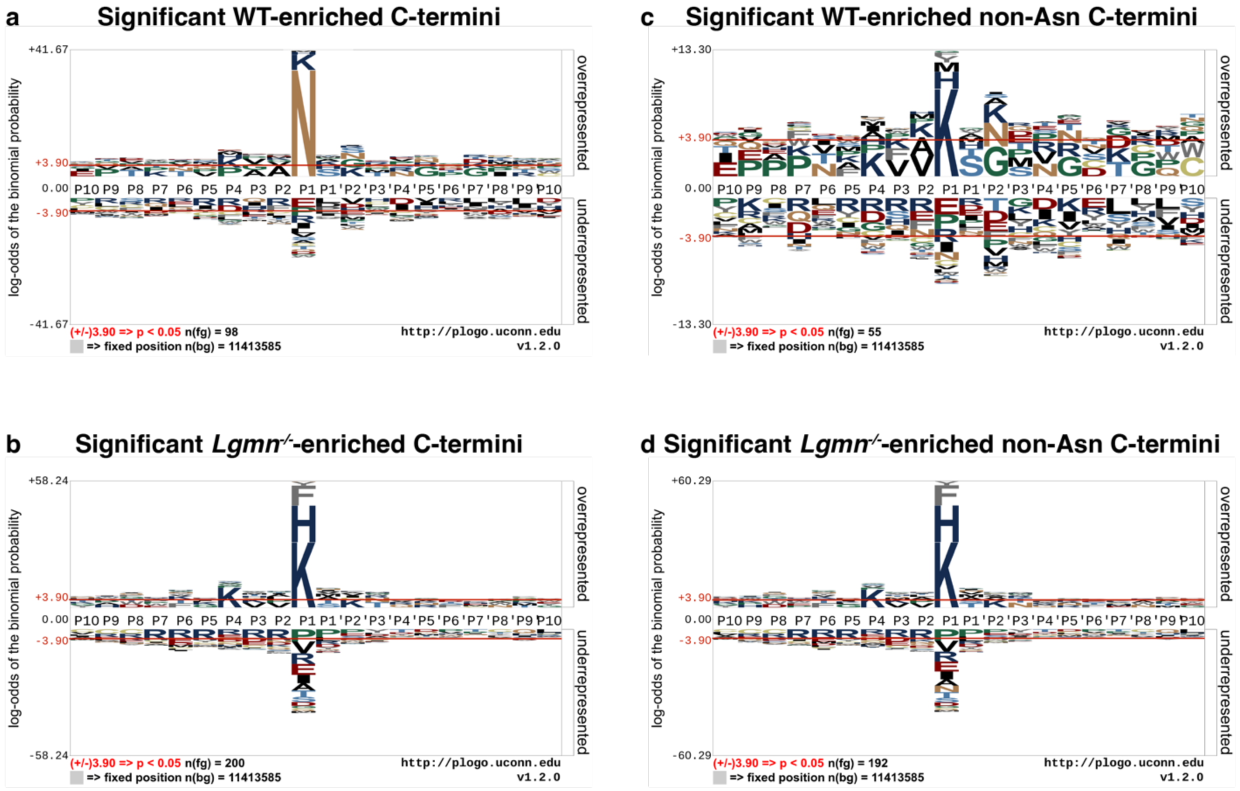

Fig. S19. Native and neo-C-termini show similar cleavage motifs as identified N-termini in wild-type and legumain deficient (*Lgmn*$^{-/-}$) murine spleen. a-d. Sequence motifs of neo-C-termini significantly enriched in WT (**a**) and *Lgmn*$^{-/-}$ (**b**) naïve spleen lysates, and non-asparaginyl neo-C-termini significantly enriched in WT (**c**) and *Lgmn*$^{-/-}$ (**d**) naïve spleen lysates were created using plogo (O'Shea et al. 2013). Overrepresented amino acids appear above and underrepresented below the x-axis ($p < 0.05$).

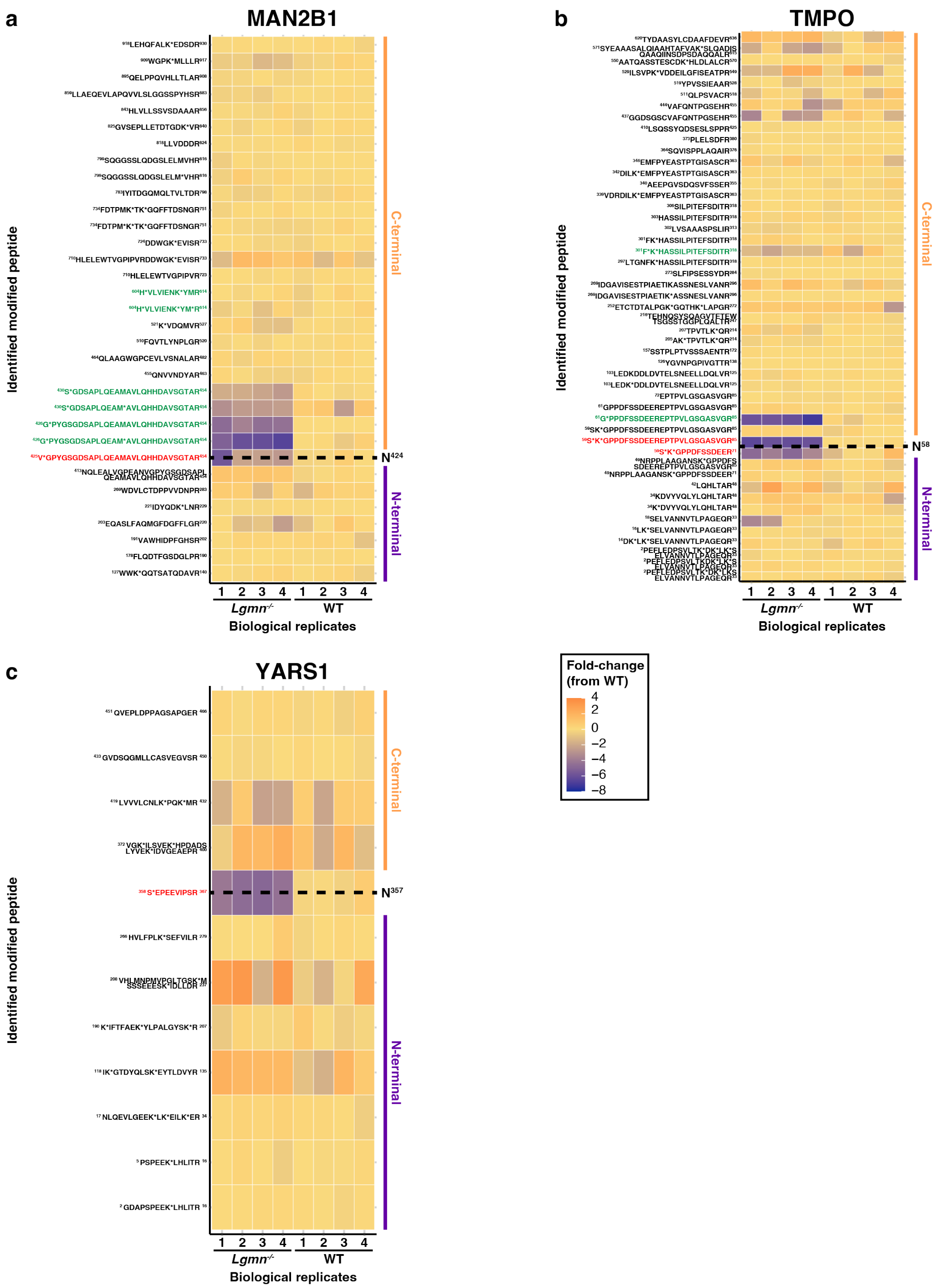

Fig. S20. Proteolytic products of identified putative legumain substrates are not differentially degraded following cleavage. a-c. Following LC-MS/MS analysis of spleen lysates (n = 4/group), data were searched against an murine proteome database in MSFragger (FragPipe v.18.0) and quantified. Max label-free quantification (LFQ) intensity values were filtered to be valid in ≥ 3 of 4 biological replicates in at least one of the groups (*Lgmn*$^{-/-}$ or WT) using Perseus (v.1.6.0.7). Fold-change of the maxLFQ intensities from the WT values for all peptides from each of MAN2B1 (**a**),

TMPO (**b**), and YARS1 (**c**) were classified as either being N-, or C-terminal to the asparaginyl cleavage sites, which are shown in red. N-termini (dimethylated N-terminal residue) are shown in green. Variable modifications including N-terminal and lysine (K) dimethylation, and methionine oxidation are indicated by an asterisk (*) in the peptide sequence. Heatmaps were visualised using the ggplot2 package in R (v.4.2.0).

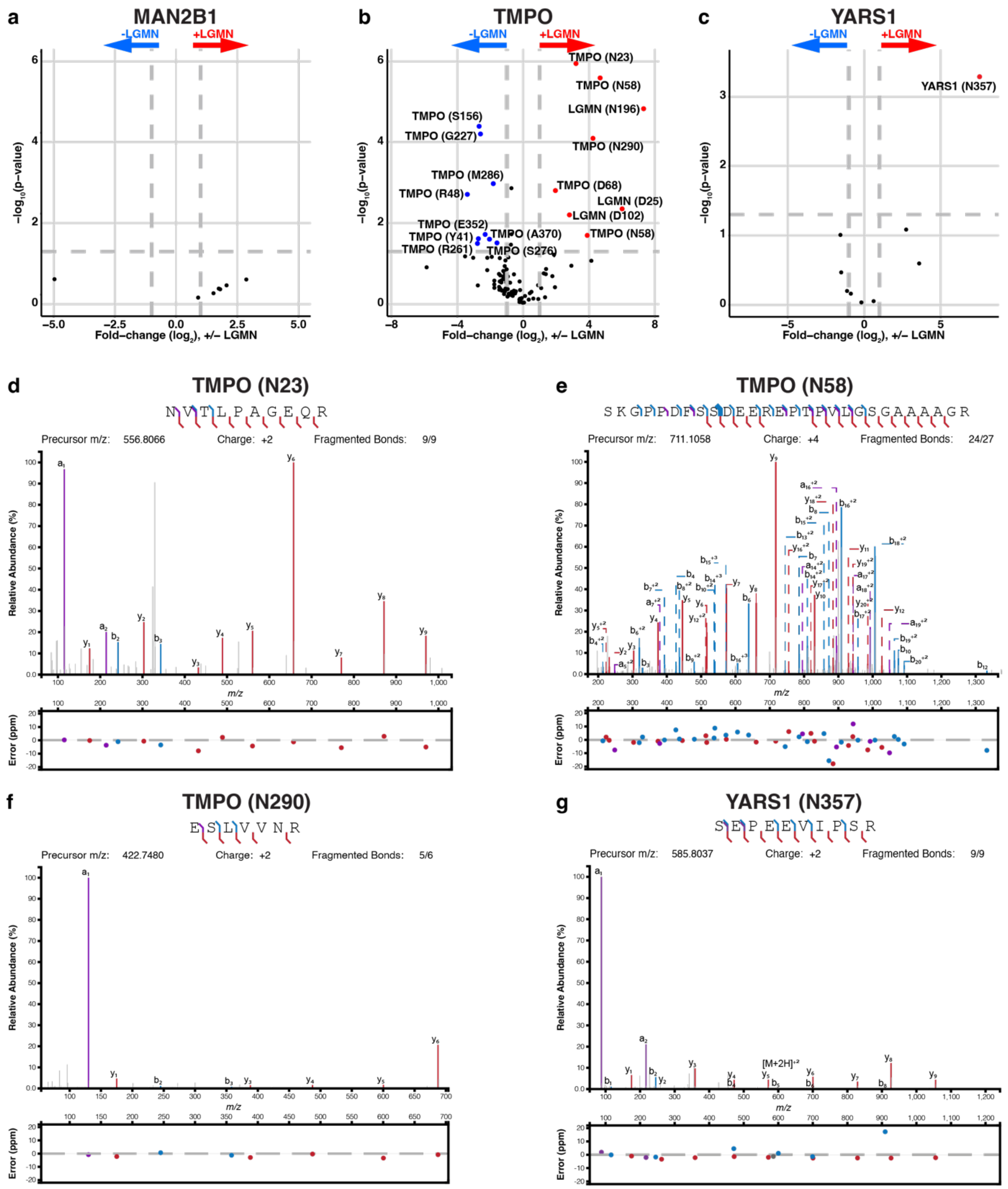

Fig. S21. Legumain directly processes various proteins *in vitro* as previously identified by FAIMS-enabled N-terminomics. a-g. Recombinant proteins were incubated with activated recombinant legumain (LGMN) prior to N-terminomics analysis (n=4/group). **a-c.** Data were filtered to contain valid quantifications in ≥ 3 of 4 replicates in at least one of the groups (+/- LGMN) and statistics were performed using Perseus (v.1.6.0.7) using a two-sample t-test. All dimethylated N-termini

were visualised by volcano plot for MAN2B1 (**a**), TMPO (**b**), and YARS1(**c**). **d-g.** MS2 analysis confirmed the dimethylation of the TMPO peptides ^{24}NVTLPAGEQR^{37}, ^{59}SKGPPDFSSDEEREPTPVLGSGAAAAGR^{86}, and ^{291}ESLVVNR^{297} and YARS1 peptide ^{358}SEPEEVIPSR^{367}within legumain-treated samples supporting their cleavage. MS/MS events were annotated with the aid of http://www.interactivepeptidespectralannotator.com/PeptideAnnotator.html with a fragment tolerance of ±20 ppm.
