# Supplementary material for: Ion Mobility–Based Enrichment-Free N-Terminomics Analysis Reveals Novel Legumain Substrates in Murine Spleen
Source: Mol Cell Proteomics. 2024 Jan 8;23(2):100714. doi: 10.1016/j.mcpro.2024.100714 (PMC10862022; doi:10.1016/j.mcpro.2024.100714)
Supplement: Supplemental Tables S33 and S34 [file mmc9.pdf]

Ion mobility-based enrichment-free N-terminomics reveal novel legumain substrates in murine spleen

Alexander R. Ziegler[1], Antoine Dufour[2,3], Nichollas E. Scott[4#], & Laura E. Edgington-Mitchell[1#]

[1]Department of Biochemistry and Pharmacology, Bio21 Molecular Science and Biotechnology Institute, The University of Melbourne, Parkville, Victoria 3052, Australia.

[2]Department of Physiology and Pharmacology, University of Calgary, Calgary, Alberta T2N 1N4, Canada.

[3]McCaig Institute for Bone and Joint Health, University of Calgary, Calgary, Alberta T2N 1N4, Canada.

[4]Department of Microbiology and Immunology, Peter Doherty Institute, The University of Melbourne, Parkville, Victoria 3052, Australia.

Table S33. Recombinant Proteins used for the *in vitro* cleavage assays.

Protein (*Gene*)	Species	Source	Catalogue Number
Legumain *(Lgmn)*	Human	N/A	Gift from Brandstetter Lab
Cathepsin S *(Ctss)*	Human	Mouse myeloma cell line	R&D, 1183-CY-010
Lysosomal alpha-mannosidase *(Man2b1)*	Human	Human embryonic kidney (HEK) cell line	R&D, 9826-GH-050
Lamina-associated polypeptide 2 *(Tmpo)*	Human	Human embryonic kidney (HEK293T) cell line	BosterBio, PROTP42167
Tyrosyl-tRNA synthetase 1 *(Yars1)*	Human	*E.coli*	BosterBio, PROTP54577

Table S34. Parallel reaction monitoring peptide inclusion list for *in vitro* cleavage assay N-terminomics analysis.

Protein (*Gene*)	Sequence	Charge	m/z
Cathepsin S *(Ctss)*	RILPDSVDWR	2	642.85010
Cathepsin S *(Ctss)*	RILPDSVDWR	3	428.90030
Cathepsin S *(Ctss)*	NITYKSNPNRILPDSVDWR	2	1158.602
Cathepsin S *(Ctss)*	NITYKSNPNRILPDSVDWR	3	772.7349
Cathepsin S *(Ctss)*	NITYKSNPNRILPDSVDWR	4	579.8013
Lysosomal alpha-mannosidase *(Man2b1)*	VGPYGSGDSAPLNEAMAVLQHHDAVSGTSR	3	1023.15
Lysosomal alpha-mannosidase *(Man2b1)*	VGPYGSGDSAPLNEAMAVLQHHDAVSGTSR	4	767.6124
Lysosomal alpha-mannosidase *(Man2b1)*	LSYNFLQVCNQLEALVGLAANVGPYGSGDSAPLNEAMAVLQHHDAVSGTSR	2	2679.295
Lysosomal alpha-mannosidase *(Man2b1)*	LSYNFLQVCNQLEALVGLAANVGPYGSGDSAPLNEAMAVLQHHDAVSGTSR	3	1786.53
Lysosomal alpha-mannosidase *(Man2b1)*	LSYNFLQVCNQLEALVGLAANVGPYGSGDSAPLNEAMAVLQHHDAVSGTSR	4	1340.148
Lamina-associated polypeptide 2 *(Tmpo)*	SKGPPDFSSDEER	2	753.85070
Lamina-associated polypeptide 2 *(Tmpo)*	SKGPPDFSSDEER	3	502.90070
Lamina-associated polypeptide 2 *(Tmpo)*	PPLPAGTNSKGPPDFSSDEER	2	1113.531
Lamina-associated polypeptide 2 *(Tmpo)*	PPLPAGTNSKGPPDFSSDEER	3	742.6875
Lamina-associated polypeptide 2 *(Tmpo)*	PPLPAGTNSKGPPDFSSDEER	4	557.2658
Tyrosyl-tRNA synthetase 1 *(Yars1)*	SEPEEVIPSR	2	585.79720
Tyrosyl-tRNA synthetase 1 *(Yars1)*	SEPEEVIPSR	3	390.86510
Tyrosyl-tRNA synthetase 1 *(Yars1)*	EKFNTPALKKLASAAYPDPSKQKPMAKGPAKNSEPEEVIPSR	2	2373.81
Tyrosyl-tRNA synthetase 1 *(Yars1)*	EKFNTPALKKLASAAYPDPSKQKPMAKGPAKNSEPEEVIPSR	3	1582.874
Tyrosyl-tRNA synthetase 1 *(Yars1)*	EKFNTPALKKLASAAYPDPSKQKPMAKGPAKNSEPEEVIPSR	4	1187.406
